# DLP 4D Printing of Programmable Molecularly‐Engineered Liquid Crystal Elastomer Actuators

**DOI:** 10.1002/advs.202517605

**Published:** 2026-01-07

**Authors:** Rakine Mouhoubi, Vincent Lapinte, Sébastien Blanquer

**Affiliations:** ^1^ Institut Charles Gerhardt Montpellier (ICGM), CNRS Université de Montpellier, ENSCM Montpellier France

**Keywords:** chain extension, digital Light Processing, LCE, two‐stage crosslinking

## Abstract

Liquid crystal elastomers (LCE) are highly attractive for 4D printing due to their ability to undergo large, rapid, and reversible shape changes in response to external stimuli. While direct ink writing (DIW) enables mesogen alignment during extrusion, it remains limited in resolution and geometric complexity. In contrast, digital light processing (DLP) offers fast, high‐resolution fabrication of complex architectures but lacks an intrinsic mechanism for aligning mesogens, which prevents reversible actuation. Here, we present a scalable and versatile strategy for DLP 4D printing of LCEs, based on partially cured printed structures subjected to mechanical programming followed by photo‐crosslinking to fix mesogen alignment. This two‐stage photo‐crosslinking approach enables the fabrication of monodomain nematic LCEs with tunable thermo‐mechanical properties and programmable, multimodal, and large actuation strains up to 45%. The strategy is demonstrated through complex LCE architectures, including an octopus model that undergoes consistent, reversible actuation over 100 thermal cycles. Additionally, sports‐themed stickman models based on these LCEs show how a single printed object can be programmed with different actuation modes, such as bending, twisting, or contraction, and their combinations, by selecting the ink best suited to the targeted actuation. These results highlight the design and programming flexibility of the method, establishing DLP as a compelling alternative to DIW for fabricating functional soft actuators.

## Graphical abstract

We introduce an original and versatile approach for printing liquid crystal elastomers that exhibit reversible shape morphing using digital light processing. 3D‐tructures are printed in a partially cured state, mechanically programmed, and then fully crosslinked to fix molecular alignment. This approach enables high‐resolution, free‐form architectures with programmable, multimodal, and large‐deformation motion, expanding the design freedom of reversible soft actuators.

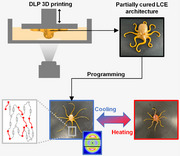

## 1 Introduction

Four‐dimensional printing extends 3D printing by enabling printed objects to change shape, properties, or function over time in response to external stimuli such as heat, light, moisture, or pH [1]. This is achieved by combining smart materials with additive manufacturing, along with carefully designed geometries and control of material response [2]. Smart materials can perform a set of elementary functions such as bending, folding, twisting, expansion or contraction, property changes, and phase transitions [3]. Understanding and combining these functions is essential for engineering deployable and reconfigurable systems used in fields such as soft robotics and biomedical devices [4]. A promising approach to achieve such transformations is to program anisotropic responses within the printed smart material, enabling global shape changes through localized control of material behavior under external stimuli.

Among the smart materials investigated for 4D printing, liquid crystal elastomers (LCEs) stand out due to their unique ability to undergo large, rapid, and reversible shape changes in response to stimuli such as heat or light [5]. LCEs combine the anisotropic molecular order of liquid crystal molecules, called mesogens, with the elasticity of a crosslinked polymer network, resulting in materials capable of programmable deformation. When heated, the mesogens undergo a nematic‐to‐isotropic transition, from an ordered to a disordered state, triggering reversible macroscopic shape changes such as bending, twisting, or linear contraction, depending on their alignment [6]. To 4D print LCEs, direct ink writing (DIW) has become a widely used technique, as it enables mesogen alignment through shear forces during extrusion [7]. DIW is a versatile method that can lead to complex deformations with spatially controlled alignment [8, 9, 10, 11, 12, 13, 14, 15]. However, most printed structures remain limited to flat geometries that fold or curve into 3D shapes. While some recent approaches have enabled DIW printing using embedded media [16] or support structures [17] to increase design freedom, these methods still fall short of achieving fully free‐form geometries. In addition to these geometric limitations, DIW‐printed structures generally lack the resolution required for detailed designs.

In contrast, digital light processing (DLP) is a widely used vat photo‐polymerization technique that enables high‐resolution printing of complex structures by photo‐crosslinking successive layers of a liquid or viscous prepolymer ink [7]. However, DLP does not enable mesogen alignment, resulting in LCEs that cannot exhibit reversible actuation. This limitation arises because DLP lacks any inherent mechanism to align mesogens, which typically results in polydomain rather than monodomain LCEs. In fact, the first reported use of DLP for fabricating LCEs involved the printing of polydomain LCEs designed for energy‐dissipative applications such as healthcare technologies, tissue replacement, and protective devices [18, 19]. Moreover, DLP printable inks also require solvents, which can disrupt liquid crystalline order [20]. The main challenge in 4D printing of LCEs using DLP 3D printing is therefore to generate structures that are complex, capable of reversible shape change, achieve large actuation strains, and perform multiple actuation modes. Addressing all these requirements is essential for enabling more sophisticated 3D shape transformations and unlocking functional LCE architectures previously inaccessible using DIW.

To our knowledge, only a few studies since 2021 have aimed to use DLP to fabricate LCE actuators, trying to overcome the inherent challenge of mesogen alignment. Two of these reported DLP 3D printing of complex polydomain LCE structures that change shape under light or heat, after being programmed by mechanical stretching [21, 22]. However, the actuation was limited to one‐way shape‐memory, with no reversibility. A solution to induce reversibility involves modifying the DLP setup. A study introduced a custom DLP printer that shears through the build platform the LC ink before photo‐polymerization, promoting mesogen alignment [23]. However, only bending actuation was shown, and it was limited to around twenty layers. Two other studies combined a magnetic field with DLP to fabricate complex LCE free‐forms with controlled mesogen orientation [24, 25]. While this approach achieved the most advanced shape programming of LCEs seen using DLP to date, the printing speed was limited and the method remained restricted to low molar mass mesogenic units, which can reduce actuation, since long oligomers are challenging to align [26]. Two other studies demonstrated the DLP 3D printing of reprogrammable LCE structures through either a shape‐memory mechanism [27] or solvent‐assisted programming [28], leading to free‐form geometries with multiple actuation modes by mechanical programming. However, although the structures were reprogrammable, they had different limitations. In the shape‐memory strategy, the actuation performance was limited when transitioning from thin films to 3D‐printed structures. In the solvent‐assisted strategy, programming occured in the swollen state during solvent evaporation, which was relatively slow and required careful control over the applied stress, solvent type, and concentration, and drying conditions.

**FIGURE 1:**
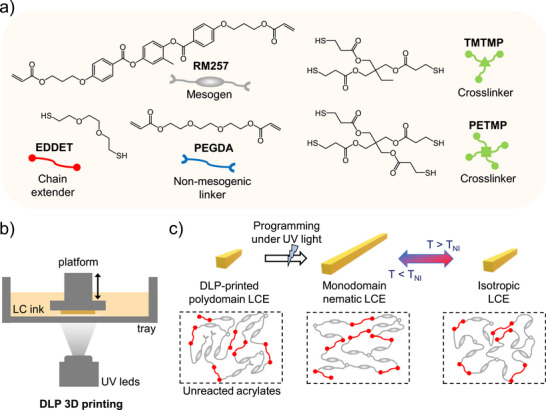
(a) Chemical compounds used in the different formulations in this study, including the mesogen, dithiol chain extender, non‐mesogenic linker, and thiol crosslinkers. (b) Schematic of the DLP 3D printing setup. (c) Conceptual illustration of the mechanical alignment process for DLP 3D printed LCEs, enabling the formation of monodomain nematic LCEs with programmable and reversible shape change.

Herein, we introduce a straightforward, versatile, and scalable method for DLP 4D printing of LCEs, enabling the fabrication of complex architectures with enhanced actuation strains, multiple programmable actuation modes, and finely tuned thermo‐mechanical properties. Controlling these properties is essential not only for actuation performance but also for selecting the most suitable LC ink for a given motion. As shown in Figure 1, partially cured DLP‐printed structures are subjected to mechanical programming followed by post‐curing to complete crosslinking and fix mesogen alignment. By fine‐tuning the synthesis strategies of LC inks, we DLP 3D print reversible shape‐changing LCEs with tunable properties and actuation responses. The potential of this approach is demonstrated through the fabrication of complex LCE free‐form architectures that exhibit reversible shape changes and multiple programmable actuation modes. From a single printed object, multiple and distinct elementary actuation modes such as bending, twisting, and linear contraction, can be programmed and combined, with the LC ink tailored to match the targeted motion. This work provides strong evidence that DLP 4D printing of LCEs is considerably promising for fabricating complex structures capable of sophisticated and reversible shape changes, and opens new possibilities for applications requiring free‐form geometries and precise actuation control.

## 2 Results and Discussion

### 2.1 General Strategy for Aligning DLP 3D‐Printed LCEs

Historically, main‐chain LCEs have been synthesized via hydrosilylation reactions, and more recently through the TAMAP approach [29, 30]. Both methods involve two‐stage crosslinking and rely on mechanical alignment during the second polymerization step. In the TAMAP approach developed by Yakacki et al., a thio‐Michael addition is first used to form a lightly crosslinked polydomain network containing excess acrylates. This network is then mechanically deformed into the desired shape, after which the remaining acrylates are photo‐polymerized under UV light to fix mesogen alignment, resulting in a monodomain LCE that can reversibly change shape.

**FIGURE 2:**
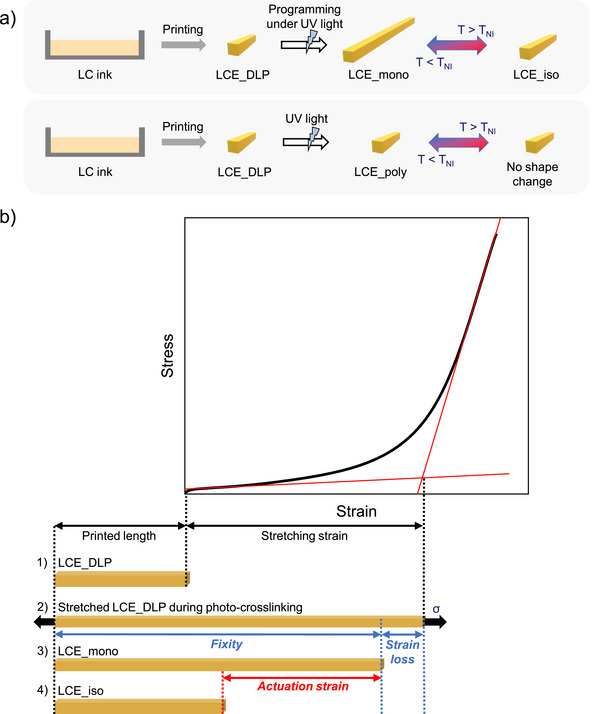
(a) Illustration of abbreviations and methods for obtaining monodomain or polydomain LCEs. (b) Schematic illustration showing the deformation evolution of a DLP‐printed LCE under a stretching programming defined by a stress‐strain curve. The scheme also shows the methodology used to calculate key strain‐related parameters, including stretching strain, fixity, and actuation strain.

Inspired by this method, we developed a strategy based on two‐stage photo‐crosslinking adapted for DLP 3D printing. This concept relies on the fabrication of LCEs with a nematic‐to‐isotropic transition temperature (T_NI_) high enough to ensure they remain in the nematic state at room temperature. The first photo‐crosslinking stage involves DLP 3D printing of LC inks to fabricate LCEs in the polydomain state. The chemical compounds used to formulate the LC inks in this study (Figure 1A) include RM257 (mesogen), EDDET (common chain extender), PEGDA (non‐mesogenic linker), TMTMP (trifunctional crosslinker), and PETMP (tetrafunctional crosslinker). The process begins with the synthesis of liquid crystal oligomers (LCO), by reacting RM257 with EDDET, which serve as the basis for all LC inks. Thio‐Michael addition was chosen as it is an efficient reaction that can be carried out under mild and environmentally friendly conditions [31]. Following formulation, the selected photo‐polymerizable LC ink is printed using a bottom‐up DLP printer equipped with a 385 nm UV light source (Figure 1b). During printing, the exposure time and light intensity are carefully controlled to maintain a consistent cured layer thickness of 100 µm, which provides good resolution while keeping the printing time reasonable. Indeed, unlike the TAMAP, the first stage of crosslinking and the fraction of unreacted acrylates are not governed by the molar ratios of the precursors but by the degree of conversion controlled through photopolymerization conditions. The resulting DLP‐printed LCE is in the polydomain state with unreacted excess acrylates (Figure 1c). The second photo‐crosslinking stage consists of post‐curing the DLP‐printed LCE under mechanical programming to fabricate a monodomain nematic LCE capable of actuation (Figure 1c). In Figure 2a, the abbreviations used throughout this study are defined. LCE_DLP corresponds to the partially cured DLP‐printed LCE. LCE_mono refers to the nematic monodomain LCE, obtained after programming and subsequent photo‐crosslinking. LCE_poly corresponds to the nematic polydomain LCE, obtained after subsequent photo‐crosslinking without prior programming.

In this study, we focus on stretching‐based programming as the representative method before investigating other actuation modes such as bending and twisting. As shown in Figure 2b, several steps are required to obtain LCE_mono through mechanical stretching programming. (1) After printing, LCE_DLP is dried without washing the remaining unreacted ink within the network. Washing would lead to a lower gel fraction after the second photocrosslinking, and the targeted properties would not be fully preserved. An uniaxial tensile test is then performed to obtain its stress‐strain curve. (2) LCE_DLP is then stretched and photo‐crosslinked under stress at a defined stretching strain, determined by the intersection of the tangent to the soft elasticity plateau and the tangent to the linear region at the end of its stress‐strain curve. In this plateau, the stress remains nearly constant with the strain, as the deformation is mainly governed by mesogen rotation. If LCE_DLP is stretched below this stretching strain, not all mesogens complete their rotation, which results in incomplete alignment, and therefore reduced actuation performance. If LCE_DLP is stretched beyond the plateau, while remaining below the failure strain, mesogens complete their rotation to the maximum extent allowed by the network. Any additional strain beyond the stretching strain then deforms the polymer network, as the mesogens can no longer be further aligned. (3) After releasing the stress, the stretched LCE sample is heated to the isotropic state and cooled back to room temperature to evaluate the fixed strain, as the LCE partially relaxes from its programmed shape. This results in LCE_mono. The fixity refers to the programmed shape retention after second photo‐crosslinking and is defined as the ratio of the fixed strain to the applied stretching strain. This indicates how faithfully the final deformation matches the imposed one, which allows to adapt or select the most suitable programming approach. (4) The resulting LCE_mono can then reversibly actuate between the nematic and isotropic states. The actuation strain is calculated as the percentage decrease in length between the nematic state at 25°C and the isotropic state at T_NI_ + 10°C, relative to the nematic length.

To evaluate the structural and thermal properties of LCEs at each step, we conducted a comprehensive set of characterization techniques, including FTIR to assess network formation, swelling experiments to estimate crosslinking density, and DSC or DMA to analyze thermal transitions and thermo‐mechanical properties. In addition, WAXS was used to evaluate mesogen alignment in monodomain LCEs, in relation to their actuation performance.

### 2.2 From Photo‐Polymerizable LC Inks to Monodomain Nematic LCEs

Three types of LC inks were investigated in this study, with the aim of understanding how to optimize our strategy and select the most suitable inks. The compositions and corresponding ink and network names are summarized in Table 1.

**TABLE 1:** Composition of LC inks expressed in molar equivalents (mol equiv.).

LC ink label	LCE network label	RM257 (mol equiv.)	PEGDA (mol equiv.)	EDDET (mol equiv.)	Crosslinker (mol equiv.)
LCO1	LCE1	1.15	—	1	—
LCO2	LCE2	1.25	—	1	—
LCO3	LCE3	1.5	—	1	—
LCO1‐PEG6.5	LCE1‐PEG6.5	1.075	0.075	1	—
LCO1‐PEG13	LCE1‐PEG13	1	0.15	1	—
LCO1‐*TMTMP*	LCE1‐*TMTMP*	1.15	—	1	0.15
LCO1‐*PETMP*	LCE1‐*PETMP*	1.15	—	1	0.15

#### 2.2.1 Synthesis, Characterization, and DLP Printing of Standard LCO Inks

**FIGURE 3:**
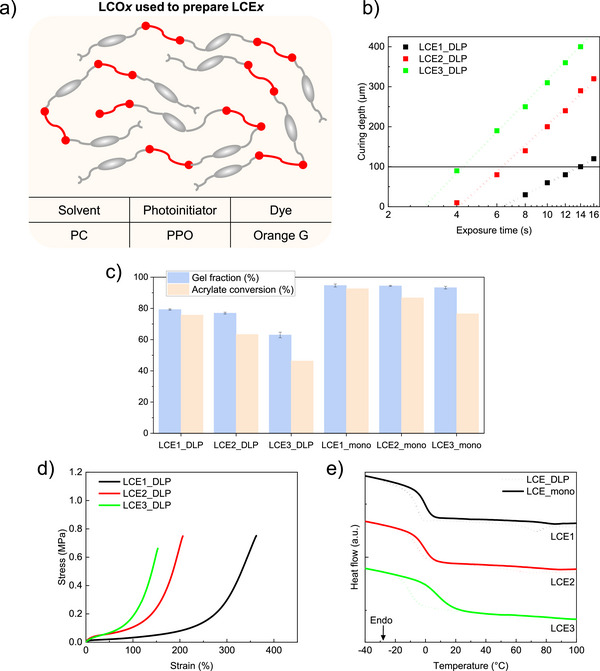
(a) Composition of LCO*x* used to prepare LCE*x*. (b) Jacob's working curves of LCO*x*, with a horizontal line at 100 µm to highlight the exposure time required to reach this cured thickness at 5 mW/cm^2^ for each ink. (c) Gel fraction and acrylate conversion of DLP‐printed LCE*x* samples and monodomain nematic LCE*x* samples. (d) Stress‐strain curve of DLP‐printed LCE*x* samples. (e) DSC thermogram of DLP‐printed LCE*x* samples (dashed lines) and monodomain nematic LCE*x* samples (solid lines). Here, *x* = 1, 2, or 3, corresponds to RM257:EDDET molar ratios 1.15:1, 1.25:1, and 1.5:1, respectively.

The first type of ink consisted of diacrylate‐terminated LCOs synthesized via base‐catalyzed thio‐Michael polyaddition, by reacting RM257 with EDDET in different molar ratios (Figure 3a). By varying the degree of polymerization (DP_n_) of LCOs, the thermo‐mechanical properties of the resulting network can be finely tuned. Diacrylate‐terminated LCOs were synthesized using a molar excess of RM257 over EDDET. The resulting inks are labeled LCO*x*, where *x* = 1, 2, or 3, corresponding to RM257:EDDET molar ratios 1.15:1, 1.25:1, and 1.5:1, respectively. LCO*x* were characterized by ^1^H NMR spectroscopy to determine DP_n_ and number‐average molar mass (M_n_) by end‐group analysis (with one repeating unit illustrated in Figure S1). Full ^1^H NMR spectra are shown in Figure S2a–c, and summarized data are provided in Table S1. DP_n_ values were determined based on the integral ratio of six protons in the diacrylate end‐groups to the four aromatic protons in the repeating mesogen unit. As expected, DP_n_ decreases from 6 to 2 as the molar ratio deviates further from stoichiometry due to the increasing excess of RM257. Ethanol‐precipitated and dried LCO*x* were then characterized by DSC to assess the thermal transitions of the LCOs. As previously reported [32], increasing LCO length leads to lower chain mobility and reduced mesogenic interactions, resulting in slightly higher T_g_ and lower T_NI_ (Figure S2d and Table S1). To fabricate DLP 3D‐printed LCE structures with excess acrylates and avoiding over‐crosslinking of layers, the layer thickness was set to 100 µm, and the optimal exposure time was determined using Jacob's equation by measuring the curing depths at 5 mW/cm^2^ (Figure 3b). Shorter LCO*x* results in faster exposure times, decreasing from 14 s for LCO1 to 4 s for LCO3. This is attributed to the higher mobility of smaller LCOs, which enhances the efficiency of photo‐polymerization.

LCO*x* were subsequently printed through the homopolymerization of acrylate end‐groups, resulting in LCE*x*_DLP samples. Gel fraction and acrylate conversion were calculated for each LCE*x*_DLP sample (Figure 3c). Acrylate conversion was determined by FTIR spectroscopy through deconvolution of the spectral region between 800 and 820 cm^−1^, focusing on the peak near 810 cm^−1^ (Figures S3–S5). The gel fraction and acrylate conversion values of the DLP‐printed LCEs are lower than fully crosslinked materials, confirming that the networks are only partially cured and still contain excess of acrylates. Nevertheless, despite gel fractions below 80%, these materials exhibit excellent mechanical integrity. Shorter LCO*x* results in a decrease in both gel fraction (from 79% to 63%) and acrylate conversion (from 76% to 46%) LCEx_DLP. This is attributed to the higher density of acrylate functional groups introduced by shorter chains. As a result, a lower acrylate conversion is sufficient to reach a 100 µm‐thick printed layer, which in turn leads to a reduced gel fraction.

The partial curing imparts similar properties across all DLP‐printed networks, regardless of the ink used. This contrasts with the behavior observed in polydomain nematic LCE*x*_poly, obtained by post‐curing LCE*x*_DLP without mechanical deformation. Unlike LCE*x*_DLP, LCE*x*_poly show efficient crosslinking with gel fractions above 90%, and follow the characteristic trends observed in LCE networks. All LCE*x*_DLP samples show comparable swelling ratios in toluene around 100%, regardless of LCO length. In contrast, LCEx_poly exhibits lower swelling ratios due to second‐stage photo‐crosslinking, with the decrease ranging from approximately 90% to 60% as the LCOs become shorter. This trend observed in LCE*x*_poly is explained by the higher number of shorter LCOs, leading to a higher concentration of acrylate groups and thus, a higher crosslinking density. As shown in Figure S6 and summarized in Table S3, DMA measurements further confirm the similarity in LCE*x*_DLP properties, with comparable glass transition temperatures (T_g, DMA_ around 0°C) and storage moduli at 25°C in the nematic state (E’_25°C_ around 0.9 MPa). In contrast, LCE*x*_poly show increasing T_g, DMA_ (from 1°C to 13°C) and E’_25°C_ (from 1 to 6 MPa) as LCO length decreases, indicating increased network stiffness due to higher crosslinking density (Figure S6 and Table S4). Higher values of E’_25°C_ and T_g,DMA_ in LCE*x*_poly compared to LCE*x*_DLP are consistent with increased crosslinking after post‐curing, and this difference becomes more pronounced as LCO length decreases. Indeed, with a gel fraction of around 63%, LCE3_DLP is the furthest from its fully crosslinked state. In contrast, this effect is minimal between LCE1_DLP and LCE1_poly, where no significant change in thermo‐mechanical properties is observed. Overall, this relatively low stiffness in the as‐printed LCE*x*_DLP, compared to their fully crosslinked counterparts, also enables greater malleability, facilitating subsequent stretching programming that will be necessary for the second‐stage photo‐crosslinking.

In order to precisely program the samples by mechanical stretching, tensile tests were performed on LCE*x*_DLP (Figure 3d and Table S3). Although the samples show similar Young moduli, both the failure strains and the extent of the soft elasticity plateau increase with LCO length, indicating that the resulting network becomes more deformable. Accordingly, the stretching strain required to align the mesogens also increases, ranging from 110% for LCE3_DLP to 285% for LCE1_DLP. After applying the programming method described earlier, the resulting monodomain nematic samples LCE*x*_mono were obtained. These samples exhibited high fixity values around 90%, with fixity exceeding 95% for LCE2_mono and LCE3_mono, showing that these networks nearly fully retain the programmed stretching strain (Table S6). As shown in Figure 3c, from LCE*x*_DLP to LCE*x*_mono, gel fraction increases by 15–30%, approaching 100%, and acrylate conversion by 17%–30% during the second photo‐crosslinking step. This confirms the formation of an additional crosslinked network that locks mesogen alignment. Regarding the evolution of thermal properties characterized by DSC, both T_g,DSC_ and T_NI,DSC_ increase after photo‐crosslinking LCE*x*_DLP into LCE*x*_mono (Figure 3e and Tables S5 and S6). This increase is attributed to the higher crosslinking density, which restricts chain mobility between segments and enhances mesogenic interactions, requiring more thermal energy to disrupt them. Furthermore, as observed for LCE*x*_poly in DMA (Figure S6 and Table S4), shorter LCOs lead to a higher T_NI,DSC_ in LCE*x*_mono (Figure 3d and Table S6). Indeed, as the proportion of mesogenic units in the network increases when using shorter LCOs, mesogenic interactions become stronger, leading to a higher T_NI_. A decrease in ΔH_NI_ is also observed after photo‐crosslinking LCE*x*_DLP into LCE*x*_mono. This can be attributed to the fact that the networks, once stretched and more densely crosslinked, not only enhance mesogenic interactions, which leads to an increase in T_NI_, but also restrict mesogen mobility, particularly during the nematic‐to‐isotropic transition. The restricted mobility imposed by these monodomain networks prevents mesogens from undergoing the transition to the same extent as in the polydomain state, resulting in less energy being absorbed during the transition.

#### 2.2.2 Synthesis, Characterization, and DLP Printing of PEG‐Containing LCO Inks

**FIGURE 4:**
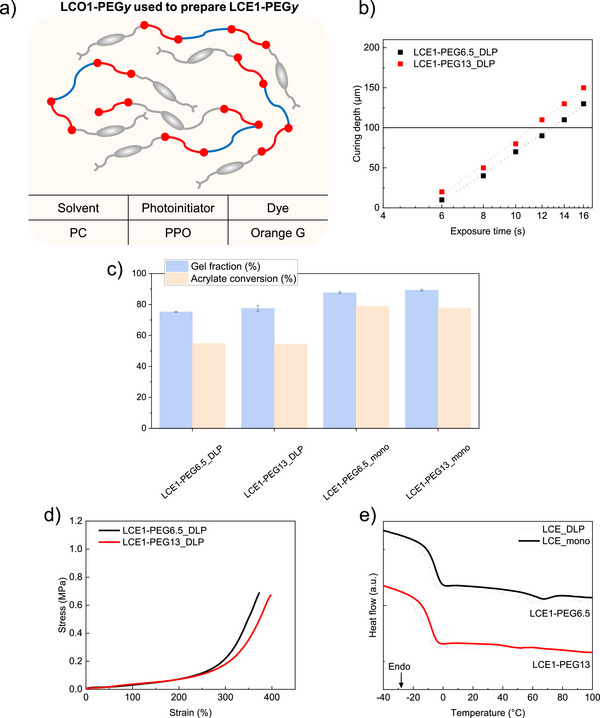
(a) Composition of LCO1‐PEG*y* used to prepare LCE1‐PEG*y*. (b) Jacob's working curves of LCO1‐PEG*y*, with a horizontal line at 100 µm to highlight the exposure time required to reach this cured thickness at 5 mW/cm^2^ for each ink. (c) Gel fraction and acrylate conversion of DLP‐printed LCE1‐PEG*y* samples and monodomain nematic LCE1‐PEG*y* samples. (d) Stress‐strain curve of DLP‐printed LCE*x* samples. (e) DSC thermogram of DLP‐printed LCE1‐PEG*y* samples (dashed lines) and monodomain nematic LCE1‐PEG*y* samples (solid lines). Here, y represents the molar percentage of RM257 substituted by PEGDA, relative to the initial RM257:EDDET ratio of 1.15:1. Accordingly, y = 6.5 or 13 for the 1.075:0.075:1 and 1:0.15:1 molar ratios, respectively.

The second type of ink consisted of PEG‐containing diacrylate‐terminated LCOs synthesized via base‐catalyzed thio‐Michael polyaddition by reacting RM257 and PEGDA with EDDET in varying molar ratios (Figure 4a). By adjusting the molar amount of PEGDA substituting RM257 in the LCOs, the thermo‐mechanical properties of the resulting network, especially the T_NI_, can be finely tuned [22]. PEG‐containing LCOs were synthesized using RM257, PEGDA, and EDDET in molar ratios of 1.075:0.075:1 and 1:0.15:1, consistently maintaining an overall molar excess of acrylate. The resulting inks are labeled LCO1‐PEG*y*, where *y* represents the molar percentage of RM257 substituted by PEGDA, relative to the initial RM257:EDDET ratio of 1.15:1. Accordingly, *y* = 6.5 or 13 for the 1.075:0.075:1 and 1:0.15:1 molar ratios, respectively. These molar ratios were selected to ensure comparable acrylate‐to‐thiol ratios, allowing a direct comparison between the networks formed from LCO1‐PEG*y* and those derived from LCO1. To confirm the incorporation of PEGDA into the repeating units of LCOs, both ^1^H NMR and DOSY NMR spectroscopies were performed on LCO1‐PEG*y*. Their spectra were compared with those of LCO*x*, and the corresponding chemical structures are shown in Figure S7. A distinct triplet signal appears in LCO1‐PEG*y*, attributed to methylene protons adjacent to the ester oxygen in the PEG‐EDDET unit, which is absent in LCO1. Using DOSY NMR spectroscopy on precipitated and dried LCO1‐PEG*y*, a similar diffusion coefficient was observed for this triplet and for signals associated with RM257‐EDDET unit, confirming that PEG is incorporated within LCO chains (Figures S8 and S9). DP_n_ for each LCO1‐PEG*y* was determined from ^1^H NMR spectra based on the combined integration of four aromatic protons from RM257‐EDDET units and four protons from PEG‐EDDET units to the combined integration of six protons from RM257‐based diacrylate end‐groups and six protons from PEG‐based diacrylate end‐groups (Figure S10a,b). A distinction can be observed in the diacrylate end‐groups, which show different signals depending on whether they originate from an RM257‐based or a PEG‐based end‐group (Figure S11). LCO1‐PEG*y* showed a DP_n_ of 9, much closer to the theoretical value of 14 predicted by Carothers’ equation, compared to LCO1 that reach a DP_n_ of 6. This higher value is attributed to the presence of PEGDA, which increases chain mobility in the reaction medium, promoting a higher DP_n_ during polyaddition (Table S2). The molar substitution of RM257 by PEGDA was quantified for both the main chains and end‐groups (Table S2). While main chain values matched the theoretical 6.5 and 13 mol%, end‐group compositions deviated, indicating PEGDA preferentially incorporates into chains rather than ends due to its higher mobility and reactivity (Figure S12). Additional explanations and calculation details are provided in the Supporting Information. The increased chain mobility introduced by PEG‐EDDET units in LCO1‐PEG*y* can be seen in the exposure times required for DLP printing (Figure 4b). LCO1‐PEG*y* exhibited shorter exposure times than LCO1, and the exposure time further decreased as the PEGDA molar content increased from LCO1‐PEG6.5 to LCO1‐PEG13. To demonstrate the effect of PEGDA addition on tuning T_NI_, DSC analysis of ethanol‐precipitated and dried LCO1‐PEG*y* shows that incorporating this non‐mesogenic linker within LCOs of the same length results in lower T_g_ and T_NI_, indicating increased chain mobility and reduced mesogenic interactions (Figure S10c and Table S2).

LCO1‐PEG*y* were subsequently printed through the homopolymerization of acrylate end‐groups, resulting in LCE1‐PEG*y*_DLP. As previously observed, these samples exhibited good mechanical integrity while maintaining gel fractions below 80% and acrylate conversion around 50% (Figures S13 and S14), indicating that the networks are partially cured (Figure 4c). DMA measurements also indicate that both LCE1‐PEG*y*_DLP networks show similar thermo‐mechanical properties in terms of T_g, DMA_ and E’_25°C_ (Figure S15 and Table S3). These properties do not change significantly after photo‐crosslinking without prior stretching in LCE1‐PEG*y*_poly, as only a slight increase in crosslinking density is observed, with swelling ratios decreasing from around 100% in LCE1‐PEG*y*_DLP to around 90% in LCE1‐PEG*y*_poly (Figure S15 and Table S4). LCE1‐PEG*y*_DLP also show similar behavior under uniaxial tensile testing, with comparable Young moduli and failure strains, leading to similar required stretching strains of around 300% (Figure 4d and Table S3). Despite slightly lower T_g,DMA_ values, attributed to PEG‐EDDET units increasing mobility of the segments within the network, LCE1‐PEG*y*_DLP exhibit overall thermo‐mechanical and mechanical properties similar to LCE1.

After applying the programming method, the resulting LCE1‐PEG*y*_mono exhibited lower fixity values than LCE*x*_mono, with values remaining sufficiently high, around 70% (Table S6). This can be attributed to the integration of PEG‐EDDET units, which reduces interactions between aligned mesogens that also help maintain the applied deformation, in addition to the newly formed crosslinking points. Furthermore, photo‐crosslinking of LCE1‐PEG*y*_DLP into LCE1‐PEG*y*_mono results in an increase of around 12% in gel fraction, approaching 90%, and around 24% in acrylate conversion, confirming the formation of an additional crosslinked network (Figure 4c). Consistent with earlier observations, DSC shows that both T_g,DSC_ and T_NI,DSC_ increase, while ΔH_NI_ decreases after photo‐crosslinking of LCE1‐PEG*y*_DLP into LCE1‐PEG*y*_mono (Figure 4e and Tables S5 and S6). Furthermore, when considering LCE1_mono in the discussion, T_NI,DSC_ decreases from 85°C to 52°C as the amount of PEGDA integrated in LCO chains increases. This decrease is attributed to the presence of these non‐mesogenic linkers, which weaken mesogenic interactions and thus lower the thermal energy required to disrupt them.

#### 2.2.3 Synthesis, Characterization, and DLP Printing of LCO Inks Using Thiol Crosslinkers

The third type of ink consisted of thiol crosslinker‐containing LC inks, labeled LCO1‐*crosslinker*, formulated by combining LCO1 with either TMTMP or PETMP, ensuring an overall molar ratio of RM257, EDDET, and the selected crosslinker of 1.15:1:0.15 (Figure S16a). This molar ratio was chosen to maintain an equimolar ratio between thiol and acrylate functional groups. This approach was investigated to explore an alternative photo‐crosslinking mechanism based on thiol‐acrylate chemistry instead of acrylate‐acrylate. For LCO1‐*crosslinker*, increasing the functionality of the thiol crosslinker led to shorter exposure times due to more frequent encounters between thiol and acrylate groups (Figure S16b). For LCE1‐*crosslinker*_DLP, increasing thiol functionality slightly increased the crosslinking density, with swelling ratios of 95% for LCE1‐*TMTMP*_DLP and 84% for LCE1‐*PETMP*_DLP. LCE1‐*crosslinker*_DLP exhibited higher gel fractions than LCE*x*‐DLP and LCE1‐PEG*y*, exceeding 85% (Figure S16c). They also showed similar thermo‐mechanical and mechanical properties, with comparable Young moduli, failure strains, stretching strains, and T_g,DMA_ (Figures S16d and S17, Table S3). Only E’_25 °C_ was slightly higher for LCE1‐*PETMP*_DLP, indicating an increase in stiffness. These properties remained nearly unchanged after photo‐crosslinking without prior stretching in LCE1‐*crosslinker*_poly, with swelling ratios decreasing only slightly to 92% for LCE1‐*TMTMP*_DLP and 83% for LCE1‐*PETMP*_DLP, showing that the second crosslinking step had only a minor effect (Figure S17, Tables S3 and S4). This is further supported by the stretching programming results for LCE1‐*crosslinker*_mono, which exhibited very low fixity values below 8%, indicating that the second crosslinking step did not effectively lock mesogen alignment (Table S6). Gel fraction values also decreased by less than 5% after photo‐crosslinking LCE1‐*crosslinker*_DLP into LCE1‐*crosslinker*_mono, further supporting this observation (Figure S16c). Regarding thermal properties, a slight increase in T_g,DSC_ was observed, indicating partial formation of a secondary network. However, both ΔH_NI_ and T_NI_ increased after photo‐crosslinking, suggesting stronger mesogenic interactions and an easier transition to the isotropic state (Figure S16e and Tables S5 and S6). This behavior contrasts with the trends observed in monodomain nematic LCEs derived from the other two ink types. The high gel fraction of LCE1‐*crosslinker*_DLP, the minimal change in properties before and after the second photo‐crosslinking, the low fixity, and the increase in ΔH_NI_ collectively indicate that LCE1‐*crosslinker*_mono remain in a polydomain state where the second crosslinking step did not successfully fix mesogen alignment. This behavior can be attributed to an already high degree of crosslinking in LCE1‐*crosslinker*_DLP, which contains too few remaining free acrylate groups. Indeed, while acrylate conversion increases by around 17% from LCE1_DLP to LCE1_mono, it increases by 8%–12% from LCE1‐*crosslinker*_DLP to LCE1‐*crosslinker*_mono (Figures S18 and S19). This suggests that thiol‐acrylate photo‐crosslinking likely requires a higher degree of acrylate conversion than acrylate‐acrylate photo‐crosslinking to achieve a stable 100 µm‐thick layer, leaving fewer unreacted acrylate groups available for forming the additional network.

### 2.3 Actuation of Printed Monodomain Nematic LCEs

**FIGURE 5:**
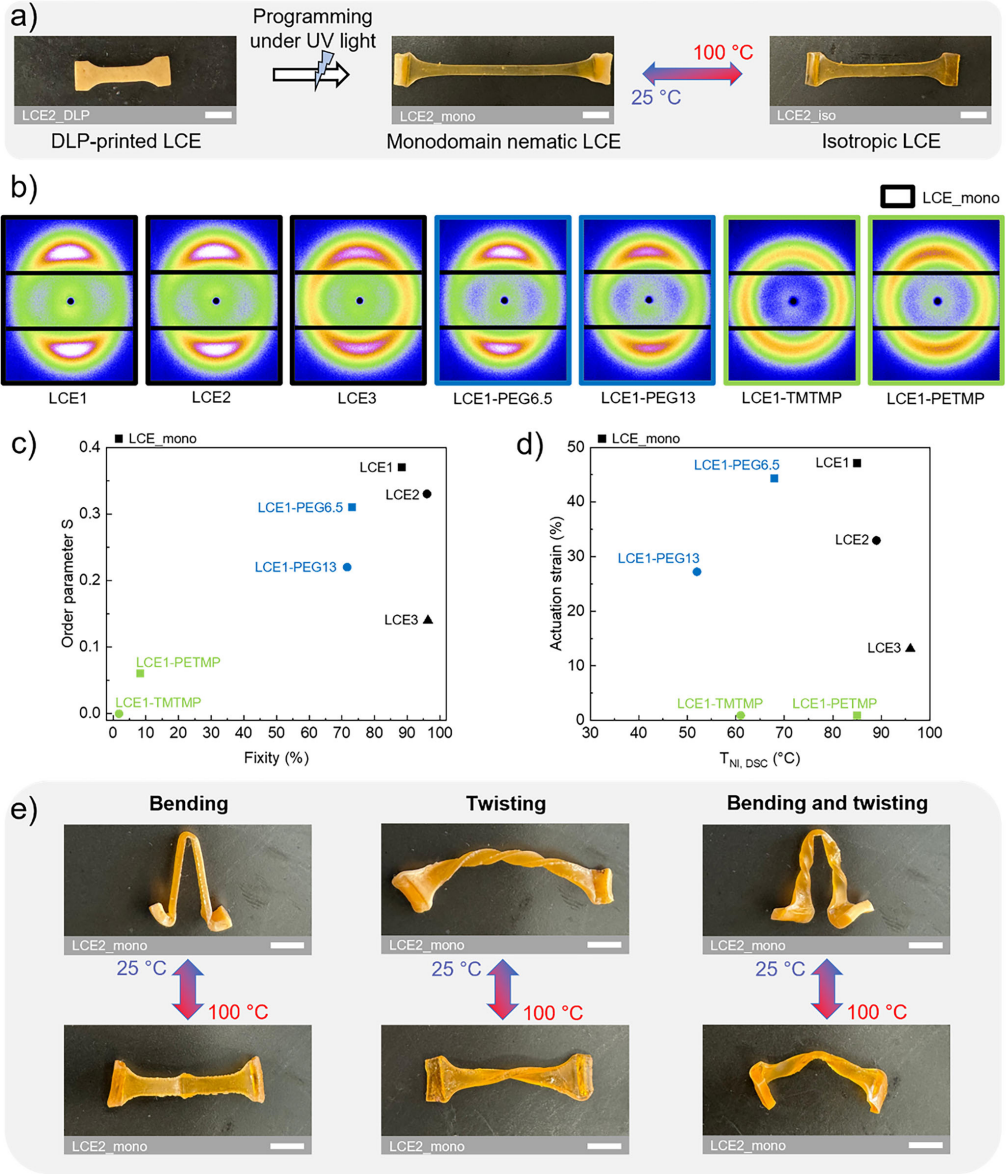
(a) Stretching programming of LCE2_DLP and thermo‐responsive reversible deformation of LCE2_mono. (b) 2D WAXS images of LCE*x*_mono, LCE1‐PEG*y*_mono and LCE1‐*crosslinker*_mono after stretching programming. (c) Order parameter as a function of fixity for LCE*x*_mono, LCE1‐PEG*y*_mono, and LCE1‐*crosslinker*_mono. (d) Actuation strain as a function of T_NI, DSC_ for LCE*x*_mono, LCE1‐PEG*y*_mono, and LCE1‐*crosslinker*_mono. (e) Thermo‐responsive reversible deformation after programming involving bending, twisting, and combined bending and twisting for LCE*x*_mono, LCE1‐PEG*y*_mono and LCE1‐*crosslinker*_mono. The white scale bars represent 0.5 cm.

In the following sections, we focus exclusively on monodomain nematic LCEs, characterizing both mesogen alignment and the resulting actuation strain (Figure 5a). The aim is to identify the most suitable network and, consequently, the optimal LC ink for the targeted motion. To distinguish and select the most suitable network for a given application, three key parameters need to be considered: actuation strain, fixity, and T_NI_.

To assess the degree of mesogen alignment in monodomain nematic LCEs, WAXS analyses were performed to calculate the order parameter *S* (Figure 5b,c). LCE1_mono, LCE2_mono and LCE1‐PEG6.5, exhibited two characteristic diffuse arcs, indicating mesogen alignment in a preferential direction. LCE3_mono and LCE1‐PEG13 showed less pronounced diffuse arcs, indicating a lower degree of mesogen alignment. LCE1‐*TMTMP*_mono and LCE1‐*PETMP*_mono exhibited an isotropic halo rather than distinct arcs, indicating a lack of preferential mesogen alignment. Order parameters calculated from the WAXS azimuthal intensity distributions further confirmed these observations with values close to zero (Figure S20), which corroborate the low fixity determined previously. The order parameter was plotted as a function of fixity for each monodomain nematic LCE (Figure 5c). *S* values for LCE1_mono, LCE2_mono and LCE1‐PEG6.5 exceed 0.3 while those for LCE3_mono and LCE1‐PEG13 are lower, at 0.14 and 0.22, respectively. These S values are comparable to those reported in the literature [25, 33]. Surprisingly, even though these network types displayed high fixity, it does not necessarily imply a high degree of mesogen alignment. When comparing LCE1_mono, LCE1‐PEG6.5_mono, and LCE1‐PEG13_mono, which have similar crosslinking densities, a trend is observed where improved fixity, driven by stronger mesogenic interactions, correlates with better mesogen alignment. However, in case of LCE1_mono to LCE3_mono, where the crosslinking density increases, this trend reverses. Despite stretching beyond the soft elasticity plateau, the increased crosslinking density limits network mobility, restricting mesogen reorientation during programming and thus reducing the degree of alignment fixed during photo‐crosslinking.

The resulting actuation strain was then determined and plotted as a function of T_NI,DSC_ for each monodomain nematic LCE (Figure 5d). This dual representation of order parameter versus fixity and actuation strain versus T_NI_ provides a comprehensive overview of the monodomain nematic LCE properties. Similar trends to those previously discussed can be observed. First, as expected, for LCE1‐*TMTMP*_mono and LCE1‐*PETMP*_mono, very low *S* values correspond to negligible actuation strain. When comparing LCE1_mono, LCE2_mono and LCE3_mono, the actuation strain decreases, following a decrease of *S* values. Likewise, when comparing LCE1_mono, LCE1‐PEG6.5_mono and LCE1‐PEG13_mono, actuation strain follows the same trend as *S*, with LCE1_mono and LCE1‐PEG6.5_mono exhibiting the highest actuation strains among all monodomain nematic LCEs, reaching values around 45%. However, a slight deviation appears when comparing LCE2_mono and LCE1‐PEG6.5_mono. While LCE2_mono has a higher *S* value, it exhibits a lower actuation strain. This is attributed to its higher crosslinking density, which restricts mesogen mobility during the nematic‐to‐isotropic transition. Therefore, it is crucial to notice that despite a higher degree of alignment, the extent of actuation is reduced due to constrained mesogen reorientation. Overall, the actuation response was relatively reproducible, with standard deviations between 1% and 3% for all samples.

In addition to stretching programming, both LCE*x*_mono and LCE1‐PEG*y*_mono are capable of undergoing elementary actuation modes such as bending and twisting, as well as combined bending‐twisting deformations (as illustrated Figure 5e and Videos S1–S3 for LCE2_mono). For these deformation modes, the same strain used for stretching is applied, after which bending or twisting is performed and fixed by the second photo‐crosslinking. Based on the results discussed above, two monodomain nematic LCEs were selected to enable the programming of distinct actuation modes in complex free‐form printed LCE structures in DLP. To DLP 3D print structures with programmed actuation modes designed for large‐amplitude motion, LCO1‐PEG6.5 was selected. This choice is based on the properties of LCE1‐PEG6.5_mono, which exhibits a high actuation strain of approximately 45%, similar to that of LCE1_mono, while offering the additional advantage of a lower T_NI_ of 68°C, which is 17°C lower than that of LCE1_mono. For the fabrication of structures requiring precise motion control, where the applied deformation needs to be closely retained, LCO2_mono was selected. This choice is based on the properties of LCE2_mono, which combines a high fixity of 96% with a suitable actuation strain of approximately 30%, making it well‐suited for applications requiring precise shape retention and controlled response. Furthermore, these two inks exhibit rheological properties compatible with DLP printing. The printable viscosity range for DLP printing typically spans from 2 × 10^2^ to 1 × 10^4^ mPa.s, ensuring good flowability and molecular diffusion. The viscosities of the two inks are approximately 2.5 × 10^3^ mPa·s for LCO1‐PEG6.5 and 6 × 10^2^ mPa.s for LCO2, both within the range of printability, and suitable for fabricating complex geometries (Figure S21).

### 2.4 DLP 3D Printing of Complex Free‐Form LCE Structures with Multiple Actuation Modes

The printable viscosity range for DLP 3D printing typically spans from 2 × 102 to 1 × 104 mPa.s, ensuring good flowability and high molecular diffusion [9]. These factors enhance reactivity during the photocuring process and facilitate uniform layer formation. Initially, PCGSu exhibited a high viscosity of approximately 3 × 104 mPa.s, whereas the formulated resins achieved a significantly lower viscosity of around 5 × 101 mPa.s, making them suitable for printing (Figure S7)

**FIGURE 6:**
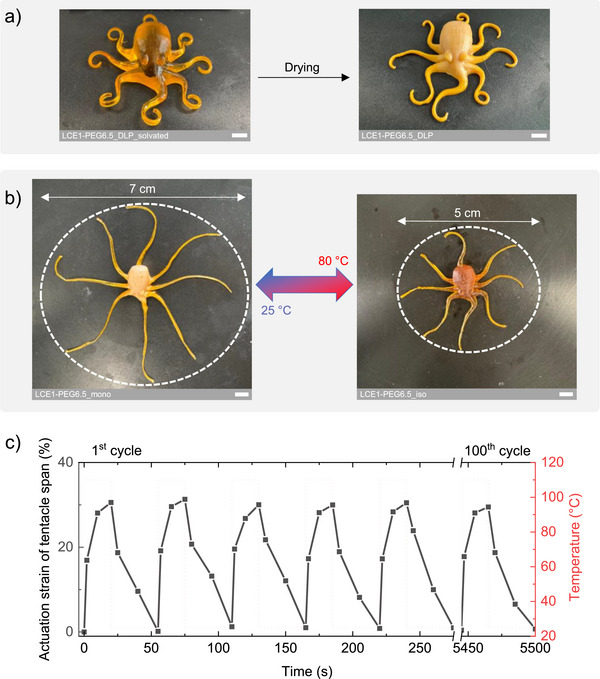
(a) DLP 3D printing and drying of an LCE octopus fabricated from LCO1‐PEG6.5. (b) Thermo‐responsive reversible deformation of a monodomain nematic LCE octopus. The dashed lines are ellipses enclosing the tentacles, representing the tentacle span in the nematic and isotropic states. (c) Cyclic actuation of the monodomain nematic LCE octopus, showing actuation strain based on tentacle span over 100 cycles. The white scale bars represent 0.5 cm.

An octopus model was first selected for DLP 3D printing as a complex LCE structure that requires high resolution and, through its tentacles, allows actuation modes targeting large deformation amplitudes. The octopus was printed using LCO1‐PEG6.5, following the previously detailed method, with high fidelity to the original STL file (Figure S22a). Printed structures before and after drying are shown in Figure 6a. The tentacles were subsequently programmed by stretching followed by a second photo‐crosslinking step. As a result, the octopus consists of a polydomain LCE1‐PEG6.5_poly head and monodomain LCE1‐PEG6.5_mono tentacles. This configuration enables the tentacles to undergo reversible actuation between the monodomain and isotropic states (Figure 6b and Video S4). Given that natural octopus arms are capable of large span variations during motion, actuation strain in the tentacle demonstrators was evaluated by measuring changes in projected span. The tentacle span exhibited an actuation strain of around 30%, characterized over a thermal actuation cycle consisting of (1) 20 s at 110°C and (2) 35 s at 25°C. Measurements were conducted during the first five cycles and at the 100^th^ cycle. The tentacle span showed highly repeatable actuation over 100 cycles (Figure 6c), with stable performance maintained over time.

In the spirit of the Paris 2024 Olympic Games and to demonstrate that multiple actuation modes can be programmed and combined within a single DLP 3D‐printed LCE architecture, we designed sports‐themed LCE stickman models to represent a boxer, a weightlifter, and a dancer. These models were printed using the previously described LCO1‐PEG6.5 and LCO2, with the printed LCE structures closely matching the original STL designs (Figure S22b). The goal was to show that from the same partially cured printed LCE architecture, it is possible to program and combine actuation modes in different ways to obtain multiple final structures with distinct and free‐form shape changes, simply by choosing the LC ink best suited to the targeted motion.

**FIGURE 7:**
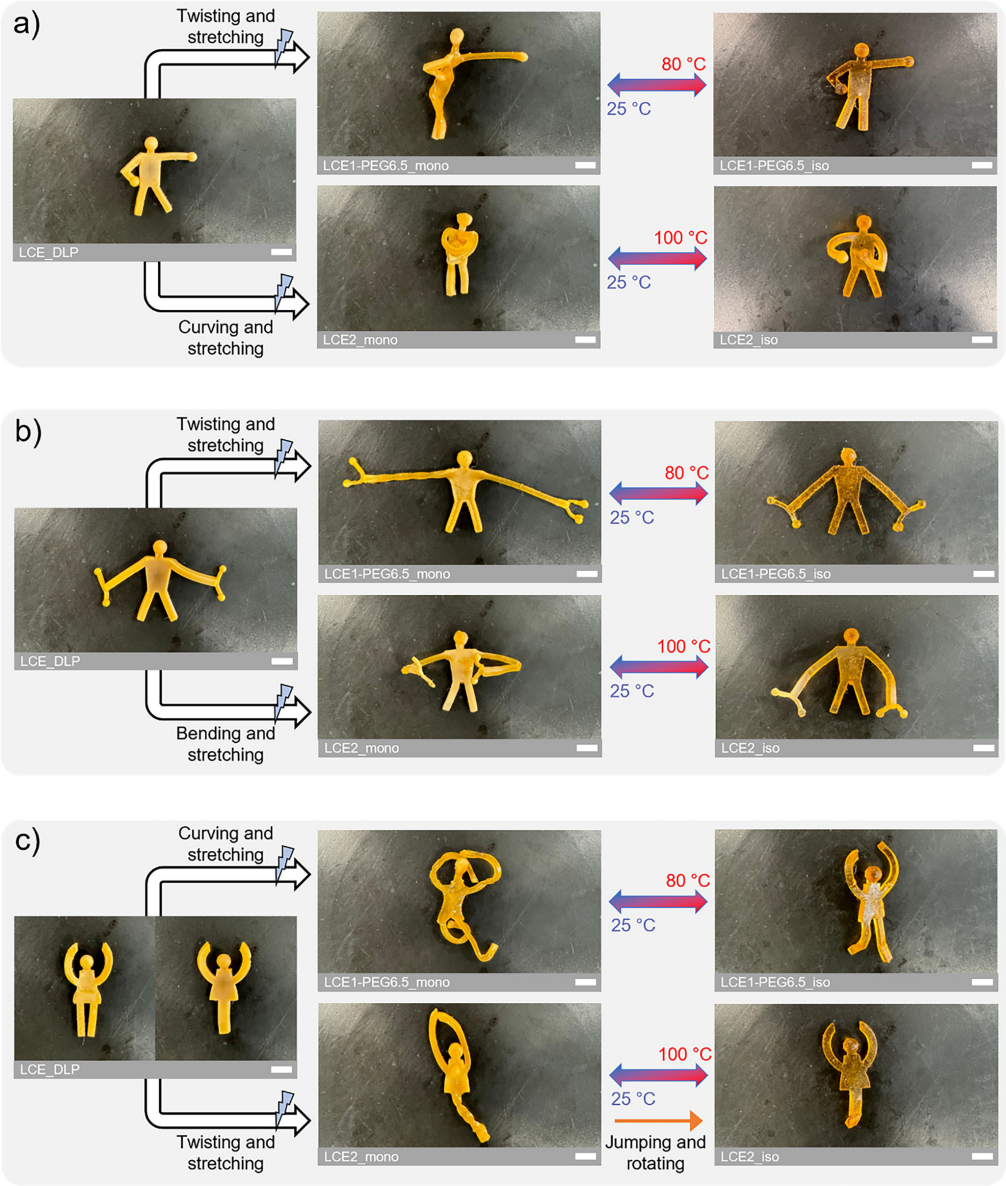
Thermo‐responsive reversible deformation behaviors after different multimodal programming for sports‐themed LCE stickman models printed from either LCO2 or LCO1‐PEG6.5: (a) boxer, (b) weightlifter, and (c) dancer. The white scale bars represent 0.5 cm.

Consequently, LCO1‐PEG6.5 was first used to program large reversible deformation. For the boxer, the body was programmed with a combination of stretching and twisting, and the arm with stretching, resulting in a reversible movement that mimics a full‐body punch (Figure 7a and Video S5). As actuation occurs in sequential steps due to the low thermal conductivity of LCEs, it can be decomposed into elementary motions, reflecting a key concept in 4D printing as introduced earlier (Figure S23a). During heating, the arm contracts first, followed by untwisting of the body. During cooling, the reverse motions occur in the same order: the arm extends first, followed by twisting of the body. This behavior is attributed to the arm being long, thinner, and mechanically isolated from the rest of the structure, allowing it to deform earlier. For the weightlifter, the arms were programmed, with one stretched and the other both stretched and twisted, to produce reversible asymmetrical arm movements that reproduce the motion of bringing weights closer to and farther from the body (Figure 7b and Video S6). For the dancer, the arms and legs were programmed with stretching and curving to emulate broad, fluid, and reversible dance‐like motions (Figure 7c and Video S7).

LCO2 was then used to program deformations with high shape retention and precise actuation, targeting motions located closer to the body. For the boxer, the arms were stretched and curved, resulting in a reversible transition from a defensive guard posture in the nematic state to an attacking stance in the isotropic state (Figure 7a and Video S8). For the weightlifter, both arms were bent, with one arm bent at a larger angle, mimicking a reversible lifting action involving asymmetric bending and straightening (Figure 7b and Video S9). As with the previous boxer, this actuation can be decomposed into elementary motions (Figure S23b). During heating, the arms first straighten, followed by contraction of the arms and the shoulders. During cooling, the reverse motions occur in the same order: the arms bend first, followed by extension of the arms and shoulders. This behavior is attributed to the fact that bending and straightening involve more localized mesogen rearrangement, requiring less collective molecular order‐disorder transition compared to full contraction. For the dancer, the legs were joined into a tail programmed with a twisting motion, while the arms were stretched. This configuration enabled the dancer to perform a jumping motion during heating, powered by the rotation of its twisted tail and accompanied by arm contraction, which could be retriggered after cooling (Figure 7c and Videos S10 and S11).

**FIGURE 8:**
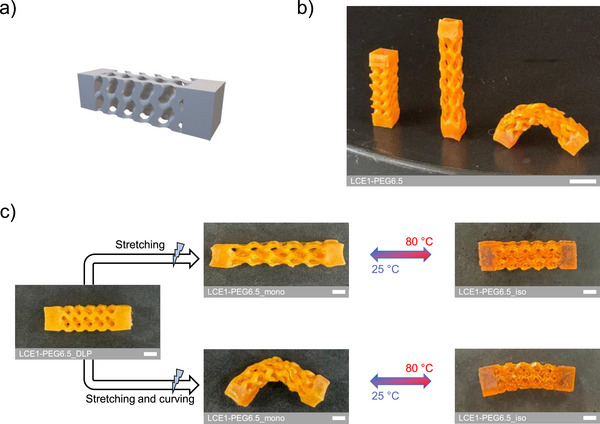
Thermo‐responsive reversible deformation behaviors of gyroids fabricated from LCO1‐PEG6.5, with actuation occurring throughout the entire architecture. a) STL file used to print the gyroid model. b) Photograph of the different gyroids from left to right: post‐printed gyroid LCE in the polydomain state, mechanically programmed stretched gyroid LCE, and mechanically stretched and curved gyroid LCE. The white scale bar represents 0.5 cm. c) Thermo‐responsive reversible deformation of the mechanically programmed monodomain nematic gyroid LCEs. The white scale bars represent 0.2 cm.

Finally, to show that the approach is also compatible with more complex 3D geometries with actuation applied throughout the structure, a gyroid model was printed (Figure 8a). The gyroid, fabricated from LCO1‐PEG6.5, was then mechanically programmed by stretching, and by stretching combined with curving, to obtain different deformation modes starting from the polydomain state (Figure 8b). This shows that beyond the actuation of simple structures shown previously, even the complex gyroid connections can be programmed, leading to a coordinated motion throughout the entire architecture. As for the octopus and the sport‐themed stickmen, reversible deformations were observed during heating, with linear contraction and straightening from the bent state (Figure 8c).

## 3 Conclusion

We introduce a straightforward, versatile, and scalable strategy for DLP 4D printing of LCEs, enabling the fabrication of complex free‐form architectures with enhanced actuation strains (up to 45%), finely tuned thermo‐mechanical properties, and multiple programmable actuation modes. This method stands as a promising alternative to DIW, overcoming limitations in design freedom and resolution by adapting the widely used TAMAP approach to DLP printing. Our approach relies on partially cured DLP‐printed structures subjected to mechanical programming, followed by a photo‐crosslinking step to fix mesogen alignment. It is first shown that achieving a monodomain nematic state requires a sufficient concentration of unreacted acrylates to form an additional network that locks the programmed mesogen orientation. Acrylate‐acrylate photo‐crosslinking proved effective, while LCEs derived from thiol‐acrylate chemistry failed to reach proper alignment, resulting in poor fixity. We have demonstrated that through precise control of the chemistry, we can both induce and understand the mechanisms governing efficient strain actuation. It is further shown that high fixity does not necessarily correspond to a high degree of alignment, and that a high degree of mesogen alignment does not automatically result in large actuation strains. This is because high crosslinking density can hinder mesogen mobility during both programming and actuation, ultimately reducing actuation performance. The comparison of these three complementary studies has made it possible to highlight differences in fixity, mesogen alignment, and actuation, which could be broadly useful for developing LCEs using two‐stage crosslinking. Based on these results, two LC inks were selected to best match the requirements for either large‐amplitude deformation or precise shape retention. To demonstrate the potential of this strategy, we DLP 3D‐printed free‐form LCE architectures, including an actuating octopus that maintains consistent and reversible deformation over 100 cycles, with an actuation strain of tentacle span of around 30%. DLP‐printed sports‐themed LCE stickmen (boxer, weightlifter, and dancer) also illustrate how a single printed architecture can be programmed to exhibit different actuation behaviors such as linear contraction, twisting, bending, curving, and their combinations, by tailoring the ink formulation and localized programming strategy. In addition, a gyroid was programmed to show reversible deformation throughout its entire 3D architecture, confirming that the method extends beyond simple demonstrators to complex geometries. These examples highlight not only the versatility of our approach but also the flexibility it offers in both design and programming. Overall, this work provides a scalable strategy to fabricate functional LCEs with programmable, multimodal, and reversible actuation, opening new opportunities for soft actuators and adaptive systems requiring complex 3D architectures with tailored stimulus responses.

## Conflicts of Interest

The authors declare no conflicts of interest.

## Supporting information




**Supporting File 1**: advs73575‐sup‐0001‐SuppMat.docx

**Supporting File 2**: advs73575‐sup‐0002‐Videos.zip

## Data Availability

The data that support the findings of this study are available from the corresponding author upon reasonable request.;

## References

[1] S. Tibbits , “4D Printing: Multi‐Material Shape Change,” Architectural Design 84 (2014): 116–121, 10.1002/ad.1710.

[2] F. Demoly , M. L. Dunn , K. L. Wood , H. J. Qi , and J.‐C. André , “The Status, Barriers, Challenges, and Future in Design for 4D Printing,” Materials & Design 212 (2021): 110193, 10.1016/j.matdes.2021.110193.

[3] S. Dimassi , F. Demoly , C. Cruz , et al., “An Ontology‐based Framework to Formalize and Represent 4D Printing Knowledge in Design,” Computers in Industry 126 (2021): 103374, 10.1016/j.compind.2020.103374.

[4] A. Ahmed , S. Arya , V. Gupta , H. Furukawa , and A. Khosla , “4D printing: Fundamentals, Materials, Applications and Challenges,” Polymer 228 (2021): 123926, 10.1016/j.polymer.2021.123926.

[5] Z.‐C. Jiang , Q. Liu , Y.‐Y. Xiao , and Y. Zhao , “Liquid Crystal Elastomers for Actuation: A Perspective on Structure‐property‐function Relation,” Progress in Polymer Science 153 (2024): 101829, 10.1016/j.progpolymsci.2024.101829.

[6] K. M. Herbert , H. E. Fowler , J. M. McCracken , K. R. Schlafmann , J. A. Koch , and T. J. White , “Synthesis and Alignment of Liquid Crystalline Elastomers,” Nature Reviews Materials 7 (2021): 23–38, 10.1038/s41578-021-00359-z.

[7] Z. Guan , L. Wang , and J. Bae , “Advances in 4D Printing of Liquid Crystalline Elastomers: Materials, Techniques, and Applications,” Materials Horizons 9 (2022): 1825–1849, 10.1039/D2MH00232A. 35504034

[8] M. O. Saed , C. P. Ambulo , H. Kim , et al., “Molecularly‐Engineered, 4D‐Printed Liquid Crystal Elastomer Actuators,” Advanced Functional Materials 29 (2019): 1806412, 10.1002/adfm.201806412.

[9] Z. Wang , Z. Wang , Y. Zheng , Q. He , Y. Wang , and S. Cai , “Three‐dimensional Printing of Functionally Graded Liquid Crystal Elastomer,” Science Advances 6 (2020): abc0034, 10.1126/sciadv.abc0034. PMC7518867 32978149

[10] G. E. Bauman , J. M. McCracken , and T. J. White , “Actuation of Liquid Crystalline Elastomers at or below Ambient Temperature,” Angewandte Chemie International Edition 61 (2022): 202202577, 10.1002/anie.202202577. 35482590

[11] D. J. Roach , X. Kuang , C. Yuan , K. Chen , and H. J. Qi , “Novel Ink for Ambient Condition Printing of Liquid Crystal Elastomers for 4D Printing,” Smart Materials and Structures 27 (2018): 125011, 10.1088/1361-665X/aae96f.

[12] X. Song , W. Zhang , H. Liu , L. Zhao , Q. Chen , and H. Tian , “3D printing of Liquid Crystal Elastomers‐based Actuator for an Inchworm‐inspired Crawling Soft Robot,” Frontiers in Robotics and AI 9 (2022): 889848, 10.3389/frobt.2022.889848. 36035870 PMC9399622

[13] Y. Wang , R. Yin , L. Jin , et al., “3D‐Printed Photoresponsive Liquid Crystal Elastomer Composites for Free‐Form Actuation,” Advanced Functional Materials 33 (2023): 2210614, 10.1002/adfm.202210614.

[14] D. J. Roach , C. Yuan , X. Kuang , et al., “Long Liquid Crystal Elastomer Fibers with Large Reversible Actuation Strains for Smart Textiles and Artificial Muscles,” ACS Applied Materials & Interfaces 11 (2019): 19514–19521, 10.1021/acsami.9b04401. 31062572

[15] A. Kotikian , J. M. Morales , A. Lu , et al., “Innervated, Self‐Sensing Liquid Crystal Elastomer Actuators with Closed Loop Control,” Advanced Materials 33 (2021): 2101814, 10.1002/adma.202101814. 34057260

[16] L. McDougall , J. Herman , E. Huntley , et al., “Free‐Form Liquid Crystal Elastomers via Embedded 4D Printing,” ACS Applied Materials & Interfaces 15 (2023): 58897–58904, 10.1021/acsami.3c14783. 38084015 PMC10739595

[17] X. Peng , S. Wu , X. Sun , et al., “4D Printing of Freestanding Liquid Crystal Elastomers via Hybrid Additive Manufacturing,” Advanced Materials 34 (2022): 2204890, 10.1002/adma.202204890. 35962737

[18] N. A. Traugutt , D. Mistry , C. Luo , K. Yu , Q. Ge , and C. M. Yakacki , “Liquid‐Crystal‐Elastomer‐Based Dissipative Structures by Digital Light Processing 3D Printing,” Advanced Materials 32 (2020): 2000797, 10.1002/adma.202000797. 32508011

[19] C. Luo , C. Chung , N. A. Traugutt , C. M. Yakacki , K. N. Long , and K. Yu , “3D Printing of Liquid Crystal Elastomer Foams for Enhanced Energy Dissipation under Mechanical Insult,” ACS Applied Materials & Interfaces 13 (2021): 12698–12708, 10.1021/acsami.0c17538. 33369399

[20] N. A. Traugutt , R. H. Volpe , M. S. Bollinger , et al., “Liquid‐crystal Order during Synthesis Affects Main‐chain Liquid‐crystal Elastomer Behavior,” Soft Matter 13 (2017): 7013–7025, 10.1039/C7SM01405H. 28930352

[21] P. Mainik , L. Hsu , C. W. Zimmer , D. Fauser , H. Steeb , and E. Blasco , “DLP 4D Printing of Multi‐Responsive Bilayered Structures,” Advanced Materials Technologies 8 (2023): 2300727, 10.1002/admt.202300727.

[22] R. Mouhoubi , P. Dieudonné‐Georges , O. Arnould , V. Lapinte , and S. Blanquer , “Toward DLP 4D Printing of Liquid Crystal Elastomers: Tailored Properties via Non‐mesogenic Linkers,” European Polymer Journal 223 (2025): 113648, 10.1016/j.eurpolymj.2024.113648.

[23] S. Li , H. Bai , Z. Liu , et al., “Digital Light Processing of Liquid Crystal Elastomers for Self‐sensing Artificial Muscles,” Science Advances 7 (2021): abg3677, 10.1126/sciadv.abg3677. PMC8302124 34301600

[24] Y. Wang , J. An , H. Kim , et al., “Printing Mosaics of Magnetically Programmed Liquid Crystal Directors for Reversibly Morphing Soft Matter,” arXiv Preprint arXiv (2024): 06590, 10.48550/arXiv.2401.06590.

[25] J. A. Herman , R. Telles , C. C. Cook , et al., “Digital Light Process 3D Printing of Magnetically Aligned Liquid Crystalline Elastomer Free–Forms,” Advanced Materials 36 (2024): 2414209, 10.1002/adma.202414209. 39468904

[26] R. Mouhoubi , V. Lapinte , and S. Blanquer , “Programmable Liquid Crystal Elastomers via Magnetic Field Assisted Oligomerization,” Advanced Functional Materials 35 (2025): 2424400, 10.1002/adfm.202424400.

[27] G. Chen , B. Jin , Y. Shi , Q. Zhao , Y. Shen , and T. Xie , “Rapidly and Repeatedly Reprogrammable Liquid Crystalline Elastomer via a Shape Memory Mechanism,” Advanced Materials 34 (2022): 2201679, 10.1002/adma.202201679. 35357046

[28] B. Jin , J. Liu , Y. Shi , G. Chen , Q. Zhao , and S. Yang , “Solvent‐Assisted 4D Programming and Reprogramming of Liquid Crystalline Organogels,” Advanced Materials 34 (2022): 2107855, 10.1002/adma.202107855. 34808005

[29] B. Donnio , H. Wermter , and H. Finkelmann , “A Simple and Versatile Synthetic Route for the Preparation of Main‐Chain, Liquid‐Crystalline Elastomers,” Macromolecules 33 (2000): 7724–7729, 10.1021/ma0002850.

[30] C. M. Yakacki , M. Saed , D. P. Nair , T. Gong , S. M. Reed , and C. N. Bowman , “Tailorable and Programmable Liquid‐crystalline Elastomers Using a Two‐stage Thiol–acrylate Reaction,” RSC Advances 5 (2015): 18997–19001, 10.1039/C5RA01039J.

[31] D. P. Nair , M. Podgórski , S. Chatani , et al., “The Thiol‐Michael Addition Click Reaction: a Powerful and Widely Used Tool in Materials Chemistry,” Chemistry of Materials 26 (2014): 724–744, 10.1021/cm402180t.

[32] R. Mouhoubi , J. Richard , V. Lapinte , and S. Blanquer , “Influence of Network Synthesis Strategies on Liquid Crystal Elastomer Properties,” Macromolecules 58 (2025): 7823–7836, 10.1021/acs.macromol.5c01037. 41768443 PMC12451743

[33] G. E. Bauman , J. D. Hoang , M. F. Toney , and T. J. White , “Degree of Orientation in Liquid Crystalline Elastomers Defines the Magnitude and Rate of Actuation,” ACS Macro Letters 12 (2023): 248–254, 10.1021/acsmacrolett.2c00754. 36715430

