# Supplementary material for: DLP 4D Printing of Programmable Molecularly‐Engineered Liquid Crystal Elastomer Actuators
Source: Adv Sci (Weinh). 2026 Jan 7;13(14):e17605. doi: 10.1002/advs.202517605 (PMC12970219; doi:10.1002/advs.202517605)
Supplement: Supplementary file 1 — Supporting File 1: advs73575‐sup‐0001‐SuppMat.docx [file ADVS-13-e17605-s002.docx]

Supporting Information for

DLP 4D Printing of Programmable Molecularly-Engineered Liquid Crystal Elastomer Actuators

Rakine Mouhoubi ^a^, Vincent Lapinte ^a^, Sébastien Blanquer ^a,*^

^a^ Institut Charles Gerhardt Montpellier (ICGM), CNRS, Université de Montpellier, ENSCM, Montpellier, France

E-mail: [sebastien.blanquer@umontpellier.fr](mailto:sebastien.blanquer@umontpellier.fr)

*Table of Contents:*
Experimental section p.S2-S9 Supplementary figures p.S10-S27
Supplementary tables p.S28-S30 Additional explanations and calculations                   p.S31

**EXPERIMENTAL SECTION**

*Materials*

1,4-Bis[4-(3-acryloyloxypropyloxy)benzoyloxy]-2-methylbenzene ([≥](https://us.vwr.com/store/product/4539845/cyclohexane-99-acs-vwr-chemicals-bdh) 95%, RM257) was purchased from Smolecule (San Antonio, USA). Propylene carbonate ([≥](https://us.vwr.com/store/product/4539845/cyclohexane-99-acs-vwr-chemicals-bdh) 95%, PC), poly(ethylene glycol) diacrylate average M_n_ 250 g/mol (PEGDA), 2,2-(ethylenedioxy)diethanethiol ([≥](https://us.vwr.com/store/product/4539845/cyclohexane-99-acs-vwr-chemicals-bdh) 95%, EDDET), pentaerythritol tetrakis(3-mercaptopropionate) ([≥](https://us.vwr.com/store/product/4539845/cyclohexane-99-acs-vwr-chemicals-bdh) 95%, PETMP), trimethylolpropane tris(3-mercaptopropionate) ([≥](https://us.vwr.com/store/product/4539845/cyclohexane-99-acs-vwr-chemicals-bdh) 95%, TMTMP), 2,6-di-tert-butyl-4-methylphenol ([≥](https://us.vwr.com/store/product/4539845/cyclohexane-99-acs-vwr-chemicals-bdh) 99%, BHT), Orange G ([≥](https://us.vwr.com/store/product/4539845/cyclohexane-99-acs-vwr-chemicals-bdh) 80%), Sudan I ([≥](https://us.vwr.com/store/product/4539845/cyclohexane-99-acs-vwr-chemicals-bdh) 95%) and phenylbis(2,4,6-trimethylbenzoyl)phosphine oxide ([≥](https://us.vwr.com/store/product/4539845/cyclohexane-99-acs-vwr-chemicals-bdh) 97%, PPO) were purchased from Sigma Aldrich (France). Triethylamine ([≥](https://us.vwr.com/store/product/4539845/cyclohexane-99-acs-vwr-chemicals-bdh) 99%, TEA) was purchased from Carlo Erba (France). All chemicals were used as-received without further purification.

*Synthesis of LC inks*

To prepare standard LC inks, RM257 and EDDET were combined in molar ratios of 1.15:1, 1.25:1 and 1.5:1. A 1:2 wt% ratio of RM257 and propylene carbonate was added to a 100 mL round-bottom flask, along with BHT (2 wt%) and PPO (2 wt%). BHT served as a free-radical inhibitor to prevent thermal crosslinking of RM257 during heating, while PPO acted as the photoinitiator. The flask was heated to 85 °C, and the mixture was magnetically stirred until homogeneous, followed by the addition of EDDET. Subsequently, TEA (2 mol%) was added dropwise to initiate polymerization. The reaction proceeded at room temperature under continuous magnetic stirring for 24 h, resulting in an isotropic LC ink. The obtained LC inks were used without further purification and were labeled “LCO*x*”, where *x* = 1, 2, or 3, corresponding to the molar ratios 1.15:1, 1.25:1, and 1.5:1, respectively.

To prepare PEG-containing LC inks, RM257, PEGDA and EDDET were combined in molar ratios of 1.075:0.075:1 and 1:0.15:1. A 1:2 wt% ratio of RM257 and propylene carbonate was added to a 100 mL round-bottom flask, along with BHT (2 wt%) and PPO (2 wt%). A similar procedure was conducted to prepare these inks. The obtained LC inks were used without further purification and were labeled “LCO1-PEG*y*”, where *y* represents the molar percentage of RM257 substituted by PEGDA, relative to the initial RM257:EDDET molar ratio of 1.15:1.

To prepare thiol crosslinker-containing LC inks, the formulated LCO1 was combined with either TMTMP or PETMP, ensuring a global molar ratio of RM257, EDDET, and the selected crosslinker of 1.15:1:0.15. The crosslinker was directly added to LCO1, and the mixture was magnetically stirred. The obtained LC inks were used without further purification and were labeled "LCO1-*crosslinker*", where *crosslinker* corresponds to the specific thiol crosslinker used.

For standard and PEG-containing LC inks, Orange G (0.1 wt%), a photo-absorber, was added before printing to enhance printing resolution. For thiol crosslinker-containing LC inks, Sudan I (0.05 wt%), was used as a photo-absorber for the same purpose.

*DLP 3D printing of LCEs*

To fabricate 3D structures, dumbbell samples, sports-themed LCE stickman and gyroid models were designed using Rhinoceros 3D and exported as STL files. Additionally, an octopus STL model sourced from Thingiverse by DiabaseEngineer (https://www.thingiverse.com/thing:159217), was used as part of the study. The 3D structures were built by stereolithography using a commercial Asiga Max X43 machine (KREOS, France) equipped with an integrated 385 nm LED and a pixel resolution of 43 μm. The layer thickness was set to 100 μm, and the optimal exposure time was determined using Jacob’s equation^[34]^ by measuring the curing depths at 5 mW/cm^2^. After printing, the structures were dried in a vacuum oven at 90 °C for 24 h to ensure complete solvent removal. The resulting DLP-printed LCE samples were labeled “LCE*x*_DLP”, “LCE1-PEG*y*_DLP”, and “LCE1-*crosslinker*_DLP”, corresponding to structures printed from LCO*x*, LCO1-PEG*y*, and LCO1-*crosslinker*, respectively.

*DLP-printed of monodomain and polydomain nematic LCEs*

To prepare monodomain nematic LCEs, the DLP-printed LCE dumbbell samples were stretched to a strain determined by tensile testing and subsequently photo-crosslinked under stress under 365 nm UV light at an intensity of 20 mW/cm^2^ for 20 min using a UV Crosslinker Bio-Link chamber (Thermo Fisher, France). The crosslinked samples were then washed by swelling in toluene for 24 h to remove any unreacted polymer residues, followed by drying in a vacuum oven at 90 °C for 24 h to ensure complete solvent removal. The resulting monodomain nematic LCE samples were labeled “LCE*x*_mono”, “LCE1-PEG*y*_mono”, and “LCE1-*crosslinker*_mono”, corresponding to structures that were stretched and subsequently photo-crosslinked from “LCE*x*_DLP”, “LCE1-PEG*y*_DLP”, and “LCE1-*crosslinker*_DLP”. Dimensional changes in monodomain nematic LCEs were photographed and analyzed using ImageJ software. The actuation strain is calculated as the percentage decrease in length between the nematic state at 25 °C and the isotropic state at T_NI, DSC_ + 10 °C (where T_NI, DSC_ is the nematic-isotropic transition temperature determined by differential scanning calorimetry), relative to the nematic length. Complementary measurements on replicas were performed with a digital caliper to calculate standard deviations.

To prepare polydomain nematic LCEs, the DLP-printed LCE samples were photo-crosslinked directly under 365 nm UV light at an intensity of 20 mW/cm^2^ for 20 min without prior stretching. The crosslinked samples were then washed by swelling in toluene for 24 h to remove any unreacted polymer residues, followed by drying in a vacuum oven at 90 °C for 24 h to ensure complete solvent removal. The resulting polydomain nematic LCE samples were labeled “LCE*x*_poly”, “LCE1-PEG*y*_poly”, and “LCE1-*crosslinker*_poly”, corresponding to structures that were photo-crosslinked directly from “LCE*x*_DLP”, “LCE1-PEG*y*_DLP”, and “LCE1-*crosslinker*_DLP”.

*Nuclear magnetic resonance spectroscopy*

^1^H NMR and DOSY NMR spectra were recorded using a 400 MHz Bruker’s Avance Spectrometer with CDCl_3_ as deuterated solvent. The chemical shifts of protons were relative to CHCl_3_ residual in CDCl_3_ at δ = 7.26 ppm. The DP_n_ values for each LC oligomer synthesised in LCO*x* were calculated from ^1^H NMR spectra based on the ratio of six protons in the diacrylate end-groups to four aromatic protons in the repeating RM257-EDDET unit, using Equation 1:

| $\mathrm{DP}_{n}=\frac{\frac{\int_{8.05}^{8.19} I}{4}}{\frac{\int_{5.81}^{5.87} I+\int_{6.07}^{6.17} I+\int_{6.36}^{6.45} I}{6}}-1$ | (1) |
| --- | --- |

The corresponding M_n_ values were calculated using Equation 2:

| $M_{n}=\mathrm{DP}_{n}\times M_{unit}+M_{end-group}$ | (2) |
| --- | --- |

where M_unit_ and M_end-group_ are the molar masses of the repeating RM257-EDDET unit and the RM257-based end group, respectively. The molar mass of the repeating unit is 772 g/mol, while the molar mass of the end group is 589 g/mol.

To confirm the incorporation of PEGDA into the repeating units of PEG-containing LC oligomers, DOSY NMR spectroscopy was performed on LCO1-PEG*y*, precipitated in ethanol and dried for 24 h in a vacuum oven at 90 °C. The DP_n_ values for each LC oligomer synthesised in LCO1-PEG*y* were calculated from ^1^H NMR spectra based on the ratio of of the combined integration of four aromatic protons in the repeating RM257-EDDET unit and four protons in the repeating PEG-EDDET unit to the combined integration of six protons in the diacrylate end-groups from a RM257-based end-group and six protons in the diacrylate end-groups from a PEG-based end-group, using Equation 3:

| $\mathrm{DP}_{n}=\frac{\frac{\int_{8.05}^{8.19} I+\int_{4.20}^{4.25} I}{4}}{\frac{\int_{5.79}^{5.87} I+\int_{6.07}^{6.18} I+\int_{6.36}^{6.45} I}{6}}-1$ | (3) |
| --- | --- |

The diacrylate end-group signals between 5.79 and 5.87 ppm were deconvoluted with Lorentzian functions, using the software TopSpin, to distinguish between diacrylate end-groups originating from a RM257-based end-group and those from a PEG-based end-group. The integrals between 5.79 and 5.87 ppm corresponding to signals assigned to RM257-based end-groups were referred to as I_dia, RM257_. The integrals between 5.79 and 5.87 ppm corresponding to signals assigned to a PEG-based end-group were referred to as I_dia, PEG_*.* The corresponding M_n_ values were calculated using Equation 4:

$M_{n}=\mathrm{DP}_{n}\times{(\frac{\int_{8.05}^{8.19} I}{\int_{8.05}^{8.19} I+\int_{4.20}^{4.25} I}{\times M}_{RM257-EDDET unit}+\frac{\int_{4.20}^{4.25} I}{\int_{8.05}^{8.19} I+\int_{4.20}^{4.25} I}\times M}_{PEG-EDDET unit})+{(\frac{\int_{5.79}^{5.87} I_{dia, RM257}}{\int_{5.79}^{5.87} I_{dia, RM257}+\int_{5.79}^{5.87} I_{dia, PEG}}{\times M}_{RM257 end-group}+\frac{\int_{5.79}^{5.87} I_{dia, PEG}}{\int_{5.79}^{5.87} I_{dia, RM257}+\int_{5.79}^{5.87} I_{dia, PEG}}\times M}_{PEG end-group})$ (4)

where M_RM257-EDDET unit_, M_PEG-EDDET unit_, M_RM257 end-group_ and M_PEG end-group_ are the molar masses of the repeating RM257-EDDET unit (772 g/mol), the repeating PEG-EDDET unit (436 g/mol), the RM257-based end-group (589 g/mol) and the PEG-based end-group (252 g/mol), respectively.

*FTIR spectroscopy*

FTIR spectra were recorded using a ThermoScientific Nicolet iS50 spectrometer equipped with a Golden-Specac heating cell and an ATR module. Data were collected with a resolution of 4 cm^-1^ and averaged over 32 scans. The acrylate C=C vibration band (ν_acrylate_) at 810 cm^-1^ and the aromatic vibration band (ν_aromatic_) at 1604 cm^-1^ were used to calculate acrylate conversion, with the aromatic band used as a normalization band since it is not affected by photocrosslinking. The spectral region between 800 and 820 cm^-1^ was deconvoluted with a pseudo-Voigt function, using the software Fityk, to accurately identify the acrylate vibration band. The calculated intensities of these bands were reported as I_acrylate_ and I_aromatic_ for the ν_acrylate_ and ν_aromatic_ bands, respectively. The conversion rate of acrylate groups during DLP 3D printing was determined using Equation 5:

| $Conversion=\frac{\left( \frac{I_{acrylate}}{I_{aromatic}} \right)^{LC ink} - \left( \frac{I_{acrylate}}{I_{aromatic}} \right)^{LCE\_DLP}}{\left( \frac{I_{acrylate}}{I_{aromatic}} \right)^{LC ink}}$ | (5) |
| --- | --- |

where $\left( \frac{I_{acrylate}}{I_{aromatic}} \right)^{LC ink}$ and $\left( \frac{I_{acrylate}}{I_{aromatic}} \right)^{LCE\_DLP}$ are the intensity ratios between the C=C and the aromatic vibration bands for the LC ink and for the DLP-printed LCE, respectively.

The conversion rate of acrylate groups during subsequent photo-crosslinking was determined using Equation 6:

| $Conversion=\frac{\left( \frac{I_{acrylate}}{I_{aromatic}} \right)^{LC ink} - \left( \frac{I_{acrylate}}{I_{aromatic}} \right)^{LCE\_mono}}{\left( \frac{I_{acrylate}}{I_{aromatic}} \right)^{LC ink}}$ | (6) |
| --- | --- |

where $\left( \frac{I_{acrylate}}{I_{aromatic}} \right)^{LC ink}$ and $\left( \frac{I_{acrylate}}{I_{aromatic}} \right)^{LCE\_mono}$ are the intensity ratios between the C=C and the aromatic vibration bands for the LC ink and for the monodomain nematic LCEs, respectively.

*Gel fraction and swelling ratio tests*

To determine gel fractions and swelling ratios, LCE samples were dried in a *vacuum* oven at 90 °C for 24 h before the initial weight was determined (m_i_). The samples were then swelled in toluene for 24 h. They were then dried in a *vacuum* oven at 90 °C for 24 h before the dried weight was determined (m_d_). They were then swelled in toluene for 24 h and weighed in a swollen state (m_s_). The gel fraction and swelling ratio for each LCE sample was calculated using Equation 7 and Equation 8, with swelling ratios reported as mean values including standard deviations as error bars from triplicates.

| $Gel fraction=\frac{m_{d}}{m_{i}}\times100$ | (7) |
| --- | --- |
| $Swelling ratio=\frac{m_{s}-m_{d}}{m_{d}}\times100$ | (8) |

*Uniaxial tensile testing*

Uniaxial tensile tests were performed to study the mechanical properties of DLP-printed LCEs. The tensile tests were executed on an Instron 3366L5885 mechanical tester equipped with a 100 N load cell. DLP-printed dumbbell LCE samples measuring approximately 18 x 2.9 x 1.3 mm^3^ were tested at a displacement rate of 5 mm/s. The Young modulus was measured by taking the slope in the linear region of the stress-strain curve between 0 and 0.5% strain. The failure strain value was defined as the maximum strain at break. The stretching strain value, rounded to the nearest multiple of 5, was determined as the strain at the intersection of the tangent to the soft elasticity plateau and the tangent to the linear region at the end of the stress-strain curve. This stretching strain corresponds to the applied strain at which DLP-printed LCEs were stretched prior to photo-crosslinking under UV light. After stretching and photo-crosslinking at this strain, the resulting monodomain nematic LCE samples were heated to the isotropic state and then cooled back to room temperature to measure a new strain, as LCEs partially relax their programmed shape. This step effectively erased the mechanical history of the material. This relaxed strain is referred to as the fixed strain. The fixity for monodomain nematic LCEs was therefore defined as the ratio of the fixed strain to the applied strain.

*Differential scanning calorimetry*

Differential scanning calorimetry (DSC) was used to evaluate the thermal properties of LC inks, DLP-printed LCEs and monodomain nematic LCEs. DSC analyses were carried out using a Netzsch DSC 3500 Sirius. The ethanol-precipitated and dried LC inks and LCE samples with a mass between 5 and 10 mg were loaded into standard aluminium DSC pans. The samples were equilibrated at -50 °C and heated to 120 °C at a rate of 10 °C/min. They were then cooled slowly to -50 °C at a rate of 2 °C/min to allow LC self-assembly. They were then heated to 120 °C at a rate of 20 °C/min. Data were reported from the second heating scans. Data are presented for the temperature range between -40 °C and 100 °C. The glass transition temperature (T_g, DSC_) was defined at the step change in the slope of the heat flow signal. The nematic-isotropic transition temperature (T_NI, DSC_) was determined at the minimum value of the endothermic peak. The enthalpy change (ΔH_NI_) was measured by integrating the endothermic energy well of the nematic-isotropic transition.

*Dynamic mechanical analysis*

Dynamic mechanical analysis (DMA) was employed to study the thermo-mechanical properties and the phase transitions of DLP-printed LCEs and polydomain nematic LCEs. DMA tests were performed using a Mettler Toledo DMA instrument. Dumbbell LCE samples measuring approximately 18 x 2.9 x 1.3 mm^3^ were tested in tensile mode, with an active length of 10 mm. The samples were equilibrated at -50 °C. They were then subjected to a strain of 0.2% at 1 Hz and heated from -50 to 120 °C at a rate of 2 °C/min. Data are presented for the temperature range between -40 °C and 100 °C. T_g, DMA_ was defined at the maximum value of the loss tangent (tan δ) curve. T_NI, DMA_ was attributed to the lowest value of the storage modulus (E’) curve just before it rises again above T_g, DMA_. The nematic modulus (E’_25 °C_) was measured using the storage modulus value at 25 °C.

*Wide angle X-ray scattering*

Wide angle X-ray scattering (WAXS) was employed to measure the orientational order of monodomain nematic LCEs. The experiments were performed with an in-house setup at Laboratoire Charles Coulomb (L2C), CNRS, Université de Montpellier, France. A high brightness low power X-ray tube, coupled with an aspheric multilayer optic (GeniX3D from Xenocs) was employed. It delivered an ultralow divergent beam (0.5 mrad, λ=0.15418 nm). Scatterless slits were used to give a clean 0.6 mm beam diameter with a flux of 35 Mphotons/s at the sample. A transmission configuration was used and the scattered intensity was measured by a 2D “Pilatus” 300 K pixel detector by Dectris (490 x 600 pixels) with a pixel size of 172×172 µm^2^, at a distance of 100 mm from the sample for WAXS configuration. The order parameter (S) was calculated using Equation 9:

| $S=\frac{\int_{0}^{\pi/2} I(\theta)\frac{3{cos}^{2}\theta-1}{2}sin\theta d\theta}{-\frac{1}{2}\int_{0}^{\pi/2} I(\theta)sin\theta d\theta}$ | (9) |
| --- | --- |

where θ is the angle between the director and mesogenic units and I(θ) is the azimuthal intensity distribution. The calculation was carried out for θ between 0 and 90 °.

*Rheology measurements*

Shear rate sweep tests on LCO1-PEG6.5 and LCO2 were performed using an Anton Paar Rheometer MCR 302 equipped with a cone plate geometry (diameter: 25 mm, angle: 2°, cone truncation: 105 µm). Measurements were carried out at 25 °C, and the shear rate was varied from 0.1 to 100 s^−1^.

**SUPPLEMENTARY FIGURES**

**Figure S1**: Chemical structure of diacrylate-terminated LCOs.

**Figure S2**: ^1^H NMR spectra of (a) LCO1, (b) LCO2 and (c) LCO3. The integrations of six protons in the diacrylate end-groups (between 5.8 and 6.5 ppm) and four aromatic protons in the repeating RM257-EDDET unit (between 8 and 8.2 ppm) are shown for each spectrum. (d) DSC thermograms of LCOx.

**Figure S3**: FTIR spectra of LCO1, LCE1_DLP, and LCE1_mono, including a zoomed-in region between 700 and 900 cm^-1^, where the acrylate C=C vibration band at 810 cm^-1^ is highlighted. The deconvoluted region between 800 and 820 cm^-1^ is shown for each sample.

**Figure S4**: FTIR spectra of LCO2, LCE2_DLP, and LCE2_mono, including a zoomed-in region between 700 and 900 cm^-1^, where the acrylate C=C vibration band at 810 cm^-1^ is highlighted. The deconvoluted region between 800 and 820 cm^-1^ is shown for each sample.

**Figure S5**: FTIR spectra of LCO3, LCE3_DLP, and LCE3_mono, including a zoomed-in region between 700 and 900 cm^-1^, where the acrylate C=C vibration band at 810 cm^-1^ is highlighted. The deconvoluted region between 800 and 820 cm^-1^ is shown for each sample.

**Figure S6**: Storage modulus (E’) and loss tangent (tan δ) curves of (a) DLP-printed LCEx_DLP and (b) their corresponding polydomain DLP-printed LCEx_poly.

**Figure S7**: Comparison of the chemical structures and ^1^H NMR spectra of LCEx and LCEx-PEGy. The methylene protons adjacent to the ester oxygen in the PEG-EDDET unit of LCEx-PEGy, and the appearance of the corresponding triplet in the ^1^H NMR spectrum of LCE1-PEG13, in contrast to LCE1, are highlighted.

**Figure S8**: DOSY NMR spectrum of LCE1-PEG6.5. The signal corresponding to the methylene protons adjacent to the ester oxygen in the PEG-EDDET unit is highlighted in the zoomed-in region of the spectrum, with its position reflecting the associated diffusion coefficient

**Figure S9**: DOSY NMR spectrum of LCE1-PEG6.5. The signal corresponding to the methylene protons adjacent to the ester oxygen in the PEG-EDDET unit is highlighted in the zoomed-in region of the spectrum, with its position reflecting the associated diffusion coefficient.

**Figure S10**: ^1^H NMR spectra of (a) LCO1-PEG6.5 and (b) LCO1-PEG13. The integrations of six protons in the diacrylate end-groups originating from either RM257 or PEG chain end (between 5.8 and 6.5 ppm), four aromatic protons in the repeating mesogen unit (between 8 and 8.2 ppm) and four protons from the PEG-EDDET unit (between 4.2 and 4.25 ppm) are shown for each spectrum. (c) DSC thermograms of LCO1-PEGy.

**Figure S11**: Comparison of ^1^H NMR spectra of LCO3 and LCO1-PEG13 highlighting the differences in diacrylate end-group peaks between 5.8 and 6.5 ppm, corresponding to signals from either the RM257 or PEG chain end.

**Figure S12**: Deconvoluted ^1^H NMR spectra of (a) LCO1-PEG6.5 and (b) LCO1-PEG13, focusing on diacrylate end-group signals between 5.79 and 5.87 ppm. The integrals corresponding to signals assigned to RM257-based end-groups were referred to as I_dia, RM257_. The integrals corresponding to signals assigned to a PEG-based end-group were referred to as I_dia, PEG_.

**Figure S13**: FTIR spectra of LCO1-PEG6.5, LCE1-PEG6.5_DLP, and LCE1-PEG6.5_mono, including a zoomed-in region between 700 and 900 cm^-1^, where the acrylate C=C vibration band at 810 cm^-1^ is highlighted. The deconvoluted region between 800 and 820 cm^-1^ is shown for each sample.

**Figure S14**: FTIR spectra of LCO1-PEG13, LCE1-PEG13_DLP, and LCE1-PEG13_mono, including a zoomed-in region between 700 and 900 cm^-1^, where the acrylate C=C vibration band at 810 cm^-1^ is highlighted. The deconvoluted region between 800 and 820 cm^-1^ is shown for each sample.

**Figure S15**: Storage modulus (E’) and loss tangent (tan δ) curves of (a) as-printed LCE1-PEG*y*_DLP and (b) their corresponding polydomain DLP-printed LCE1-PEG*y*_poly.

**Figure S16**: (a) Composition of LCO1-*crosslinker* used to prepare LCE1-*crosslinker*. (b) Jacob’s working curves of LCO1-*crosslinker*, with a horizontal line at 100 µm to highlight the exposure time required to reach this cured thickness at 5 mW/cm^2^ for each ink. (c) Gel fractions and acrylate conversions of DLP-printed LCE1-*crosslinker* samples and monodomain nematic LCE1-*crosslinker* samples. (d) Stress-strain curves of DLP-printed LCE1-*crosslinker* samples. (e) DSC thermograms of DLP-printed LCE1-*crosslinker* samples (dashed lines) and monodomain nematic LCE1-*crosslinker* samples (solid lines).

**Figure S17**: Storage modulus (E’) and loss tangent (tan δ) curves of (a) as-printed LCE1-*crosslinker*_DLP and (b) their corresponding polydomain DLP-printed LCE1-*crosslinker*_poly.

**Figure S18**: FTIR spectra of LCO1-TMTMP, LCE1-TMTMP_DLP, and LCE1-TMTMP_mono, including a zoomed-in region between 700 and 900 cm^-1^, where the acrylate C=C vibration band at 810 cm^-1^ is highlighted. The deconvoluted region between 800 and 820 cm^-1^ is shown for each sample.

**Figure S19**: FTIR spectra of LCO1-PETMP, LCE1-PETMP_DLP, and LCE1-PETMP_mono, including a zoomed-in region between 700 and 900 cm^-1^, where the acrylate C=C vibration band at 810 cm^-1^ is highlighted. The deconvoluted region between 800 and 820 cm^-1^ is shown for each sample.

**Figure S20**: WAXS azimuthal intensity distributions of LCEx_mono, LCE1-PEGy_mono and LCE1-crosslinker_mono.

**Figure S21**: Viscosity as a function of shear rate for LCO1-PEG6.5 and LCO2. The orange region indicates the viscosity range suitable for DLP-printable inks.

**Figure S22**: STL files used for DLP 3D printing of complex 3D architectures, including (a) an octopus and (b) sports-themed stickman models. The octopus model was sourced from Thingiverse by DiabaseEngineer (<https://www.thingiverse.com/thing:159217>).

**Figure S23**: Temporal decomposition of motion during heating and cooling cycles of the sports-themed LCE stickman models, (a) boxer and (b) weightlifter, based on videos S5 and S9.

**SUPPLEMENTARY TABLES**

**Table S1**: Summary of ^1^H NMR data for LCO*x* characterization.

|  | DP_n_^a^ calc. | M_n_ calc.^b^ (g/mol) | DP_n_^c^ theo. | T_g, DSC_^d^  (°C) | T_NI, DSC_^d^  (°C) |
| --- | --- | --- | --- | --- | --- |
| LCO1 | 6 | 5500 | 14 | -7 | 73 |
| LCO2 | 4 | 3600 | 9 | -8 | 77 |
| LCO3 | 2 | 2300 | 5 | -9 | 84 |

^a^ Calculated according to Equations 1 and 3 from ^1^H NMR spectra. ^b^ Calculated according to Equations 2 and 4 from ^1^H NMR spectra. ^c^ Calculated according to Carother’s equation. ^d^ Determined by DSC.

**Table S2**: Summary of ^1^H NMR data for LCO1-PEG*y* characterization.

|  | DP_n_^a^ calc. | M_n_ calc.^b^  (g/mol) | DP_n_^c^ theo. | y_LCO_^d^  (mol%) | y_end-group_^d^  (mol%) | T_g, DSC_^e^  (°C) | T_NI, DSC_^e^  (°C) |
| --- | --- | --- | --- | --- | --- | --- | --- |
| LCO1-PEG6.5 | 9 | 7400 | 14 | 6.6 | 3.1 | -10 | 60 |
| LCO1-PEG13 | 9 | 7300 | 14 | 11.9 | 6.9 | -13 | 45 |

^a^ Calculated according to Equations 1 and 3 from ^1^H NMR spectra. ^b^ Calculated according to Equations 2 and 4 from ^1^H NMR spectra. ^c^ Calculated according to Carother’s equation. ^d^ Calculated according to Equations 5 and 6 from ^1^H NMR spectra. ^d^ Determined by DSC.

**Table S3**: Summary of tensile test and DMA data for LCE*x*_DLP, LCE1-PEG*y*_DLP and LCE1-*crosslinker*_DLP.

|  | Young modulus^a^ (10^-1^ MPa) | Failure strain^a^ (%) | Stretching strain^a^ (%) | T_g, DMA_^b^ (°C) | T_NI, DMA_^b^ (°C) | E’_25 °C_^b^ (MPa) |
| --- | --- | --- | --- | --- | --- | --- |
| LCE1_DLP | 5.7 ± 1.4 | 347 ± 16 | 285 | 1 | 72 | 0.81 |
| LCE2_DLP | 8.5 ± 1.3 | 215 ± 7 | 155 | 0 | / | 0.96 |
| LCE3_DLP | 4.5 ± 0.6 | 149 ± 7 | 110 | -1 | / | 0.97 |
| LCE1-PEG6.5_DLP | 4.6 ± 0.2 | 350 ± 21 | 285 | -2 | 60 | 0.68 |
| LCE1-PEG13_DLP | 3.4 ± 0.7 | 382 ± 14 | 315 | -5 | 40 | 0.48 |
| LCE1-*TMTMP*_DLP | 5.7 ± 1.3 | 371 ± 25 | 205 | 1 | 54 | 0.70 |
| LCE1-*PETMP*_DLP | 6.1 ± 0.7 | 325 ± 20 | 215 | 2 | 57 | 1.22 |

^a^ Determined by uniaxial tensile testing. ^b^ Determined by DMA.

**Table S4**: Summary of DMA data for LCE*x*_poly, LCE1-PEG*y*_poly and LCE1-*crosslinker*_poly.

|  | T_g, DMA_^a^ (°C) | T_NI, DMA_^a^ (°C) | E’_25 °C_^a^ (MPa) |
| --- | --- | --- | --- |
| LCE1_poly | 1 | 70 | 1.23 |
| LCE2_poly | 4 | 93 | 2.26 |
| LCE3_poly | 13 | / | 6.37 |
| LCE1-PEG6.5_poly | -2 | 65 | 0.67 |
| LCE1-PEG13_poly | -4 | 48 | 0.75 |
| LCE1-*TMTMP*_poly | 2 | 62 | 0.67 |
| LCE1-*PETMP*_poly | 2 | / | 1.24 |

^a^ Determined by DMA.

**Table S5**: Summary of DSC data for LCE*x*_DLP, LCE1-PEG*y*_DLP and LCE1-*crosslinker*_DLP.

|  | T_g, DSC_^a^  (°C) | T_NI, DSC_^a^  (°C) | ΔH_NI_^a^  (J/g) |
| --- | --- | --- | --- |
| LCE1_DLP | -5 | 73 | -1.33 |
| LCE2_DLP | -6 | 72 | -1.19 |
| LCE3_DLP | -9 | 68 | -1.13 |
| LCE1-PEG6.5_DLP | -9 | 62 | -1.29 |
| LCE1-PEG13_DLP | -13 | 42 | -0.77 |
| LCE1-*TMTMP*_DLP | -4 | 61 | -0.75 |
| LCE1-*PETMP*_DLP | 0 | 69 | -1.15 |

^a^ Determined by DSC.

**Table S6**: Summary of fixity and DSC data for LCE*x*_mono, LCE1-PEG*y*_mono and LCE1-*crosslinker*_mono.

|  | Fixity  (%) | T_g, DSC_^a^  (°C) | T_NI, DSC_^a^  (°C) | ΔH_NI_^a^  (J/g) |
| --- | --- | --- | --- | --- |
| LCE1_mono | 88 | 0 | 85 | -0.56 |
| LCE2_mono | 96 | -1 | 90 | -0.74 |
| LCE3_mono | 96 | 8 | 94 | -0.06 |
| LCE1-PEG6.5_mono | 73 | -5 | 67 | -1.16 |
| LCE1-PEG13_mono | 72 | -8 | 52 | -0.20 |
| LCE1-*TMTMP*_mono | 2 | -2 | 61 | -1.18 |
| LCE1-*PETMP*_mono | 8 | 5 | 85 | -2.31 |

^a^ Determined by DSC.

**ADDITIONNAL EXPLANATIONS AND CALCULATIONS**

**Experimental determination of the molar percentage of RM257 substituted by PEGDA**

The molar percentage of RM257 substituted by PEGDA in LCO1-PEG*y*, relative to the initial RM257:EDDET molar ratio of 1.15:1, was determined by ^1^H NMR spectroscopy for the entire oligomer (y_LCO_) and for the end-groups (y_end-group_) using Equations S1 and S2, respectively:

| $y_{\mathrm{LCO}}=\frac{\int_{4.20}^{4.25} I}{\int_{8.05}^{8.19} I+\int_{4.20}^{4.25} I}$ | (S1) |
| --- | --- |

| $y_{end-group}=\frac{\int_{5.79}^{5.87} I_{dia, PEG}}{\int_{5.79}^{5.87} I_{dia, RM257}+\int_{5.79}^{5.87} I_{dia, PEG}}$ | (S2) |
| --- | --- |

where $\int_{4.20}^{4.25} I$ corresponds to four protons in the repeating PEG-EDDET unit, $\int_{8.05}^{8.19} I$ corresponds to four aromatic protons in the repeating RM257-EDDET unit, $\int_{5.79}^{5.87} I_{dia, PEG}$ corresponds to diacrylate end-group signals assigned to PEG-based end-groups, and $\int_{5.79}^{5.87} I_{dia, RM257}$ corresponds to diacrylate end-group signals assigned to RM257-based end-groups.

The calculated values are summarized in Table S2. For y_LCO_, the experimental values closely match the theoretical values of 6.5 and 13 mol%, also indicating successful substitution of RM257 by PEGDA. In contrast, y_end-group_ showed values that deviated from the expected ones, based on deconvolution of the ^1^H NMR spectra distinguishing RM257-based from PEG-based diacrylate end-groups (Figure S12). This suggests that PEGDA is more likely to be incorporated within LCO chains than at their ends. This behavior is attributed to the higher mobility of PEGDA compared to RM257, which makes it react faster and less likely to end up at the chain ends.
